# Looking into troubled waters: Childhood emotional maltreatment modulates neural responses to prolonged gazing into one’s own, but not others’, eyes

**DOI:** 10.3758/s13415-023-01135-y

**Published:** 2023-10-25

**Authors:** Mirjam C. M. Wever, Lisanne A. E. M. van Houtum, Loes H. C. Janssen, Wilma G. M. Wentholt, Iris M. Spruit, Marieke S. Tollenaar, Geert-Jan Will, Bernet M. Elzinga

**Affiliations:** 1 Department of Clinical Psychology, Faculty of Social and Behavioral Sciences, Leiden University, 2300 RB Leiden, The Netherlands; 2 Leiden Institute for Brain and Cognition, Leiden, the Netherlands; 3 Department of Clinical Psychology, Utrecht University, Utrecht, the Netherlands

**Keywords:** Childhood emotional maltreatment, Self-referential processing, Direct gaze, Ventromedial prefrontal cortex, Eye tracking, Social cognition

## Abstract

One of the most prevalent nonverbal, social phenomena known to automatically elicit self- and other-referential processes is eye contact. By its negative effects on the perception of social safety and views about the self and others, childhood emotional maltreatment (CEM) may fundamentally affect these processes. To investigate whether the socioaffective consequences of CEM may become visible in response to (prolonged) eye gaze, 79 adult participants (mean [M]_*age*_ = 49.87, standard deviation [SD]_*age*_ = 4.62) viewed videos with direct and averted gaze of an unfamiliar other and themselves while we recorded self-reported mood, eye movements using eye-tracking, and markers of neural activity using fMRI. Participants who reported higher levels of CEM exhibited increased activity in ventromedial prefrontal cortex to one’s own, but not to others’, direct gaze. Furthermore, in contrast to those who reported fewer of such experiences, they did not report a better mood in response to a direct gaze of self and others, despite equivalent amounts of time spent looking into their own and other peoples’ eyes. The fact that CEM is associated with enhanced neural activation in a brain area that is crucially involved in self-referential processing (i.e., vmPFC) in response to one’s own direct gaze is in line with the chronic negative impact of CEM on a person’s self-views. Interventions that directly focus on targeting maladaptive self-views elicited during eye gaze to self may be clinically useful.

**Supplementary information:**

The online version contains supplementary material available at 10.3758/s13415-023-01135-y.

## Introduction

Childhood emotional maltreatment (CEM) is common with global prevalence rates between 18.4–36.3% (Stoltenborgh et al., 2015). It encompasses both emotional abuse (i.e., verbal assaults and demeaning behaviors directed towards children by adults that are harmful for a child’s self-worth) and emotional neglect (i.e., caregivers’ irresponsibility or failure in satisfying children's basic psychological needs for love, belonging, nurturance, and support) (Bernstein et al., 1994; Bernstein et al., 2003). It is considered one of the most devastating forms of maltreatment due to its chronic exposure pattern from an early age onwards and the involvement of a primary caregiver.

CEM gives rise to long-term negative consequences into adulthood (Egeland, 2009; Gilbert et al., 2009; Reyome, 2010; Spertus et al., 2003; van Harmelen et al., 2010). One of these potential outcomes is the development of negative cognitions about the self and others as a result of the perceived betrayal of trust during childhood by a primary caregiver (Baugh et al., 2019). CEM may result in the generalization of distrust to others, leading to maladaptive other-schemas (e.g., everyone has bad intentions), whereas it may also fuel maladaptive schemas about the self, leading to the belief that they are unloved, worthless, or unwanted (Baugh et al., 2019; Gobin & Freyd, 2014). Although studies, including a direct comparison between responses to the self and others are sparse, people with a history of CEM seem to be particularly vulnerable to develop negative self-views compared with other types of maltreatment (i.e., physical or sexual abuse) (Alloy et al., 2006; Gibb, 2002; Gibb, Abramson, & Alloy, 2004; Rose & Abramson, 1992; van Harmelen et al., 2010). Negative self-views put people with a history of CEM at a greater risk for developing internalizing disorders, such as anxiety disorders and depression, and can contribute to interpersonal difficulties and problems in the formation and maintenance of (intimate) relationships (Reyome, 2010; Riggs, 2010; Wright et al., 2009).

One of the most prevalent nonverbal, social phenomena known to automatically elicit both self- and other-referential processes is eye contact (Conty et al., 2016). Eye contact with others generally elicits positive feelings (Hietanen, 2018; Wever et al., 2022). However, people who were abused as a child often perceive eye contact as a signal of threat (Krill, 2011; Wilkinson, 2010). Hence, avoiding eye contact may be an automatic response in individuals with CEM as a means to cope with negative affect caused by face-to-face interactions (Tottenham et al., 2011). In addition to *social* eye contact, gazing into one’s own eyes (e.g., in the mirror) elicits strong emotional responses related to the self in people with low self-esteem, including feelings of shame and disgust (Erdem, 2019; Ypsilanti et al., 2020). However, how a history of CEM may affect the processing of gazing into one’s own eyes has yet to be determined.

While there is a dearth of studies on neural circuitry supporting self- and other-referential processing during prolonged eye gazing to the self and others, neuroimaging studies have identified a consistent network of regions that respond to static pictures or judgements of the self and others, consisting of distinct parts of the medial prefrontal cortex, insula, temporoparietal junction (TPJ), posterior cingulate cortex (PCC), and cuneus (D'Argembeau, 2013; Denny et al., 2012; Lemogne et al., 2011; Murray et al., 2012; Northoff et al., 2006; van der Meer et al., 2010). Especially the ventromedial prefrontal cortex (vmPFC) and dorsomedial prefrontal cortex (dmPFC) seem to lay at opposite ends of a functional spectrum representing the processing of affective self-relevant and other-relevant information, respectively (Denny et al., 2012).

Literature on the association between CEM and neural responses to self- and other-related content is limited to the finding that people with a history of CEM exhibited enhanced amygdala reactivity in response to neutral and emotional faces (Dannlowski et al., 2012; Tottenham et al., 2011; van Harmelen et al., 2013). Only a few studies have examined whether a history of CEM is associated with neural responses to self-related content (Puetz et al., 2021; Talmon et al., 2021), but none of them examined the relationship between a history of CEM and gazing into one’s own and others’ eyes. This is striking, because eye contact with others is fundamental to our daily lives and plays an important role in our social connections with others (Emery, 2000). In addition, connecting with ourselves (e.g., via a mirror) elicits powerful affective and physiological responses, which facilitates identification and mitigation of one’s (maladaptive) responses when being confronted with oneself (Baldwin, 1996; Vergallito et al., 2020).

To better understand processes supporting eye contact with the self and others in individuals with a history of CEM, we examined associations between self-reported CEM and participants’ mood, gaze, and neural responses to direct and averted gaze of themselves and an unfamiliar other adult. First, we expected that people reporting higher levels of CEM would report lower mood after direct (vs. averted) gaze videos and that they would gaze less often into the eyes of self and others compared with people who report lower levels of CEM. Given that no studies have examined the association between experienced CEM and people’s neural responses to gazing into one’s own and others’ eyes, our neuroimaging analyses are exploratory. Examining how individuals with a history of CEM respond to their own or other people’s direct gaze might not only yield new insights in fundamental processes of human nature but also may contribute to new interventions for individuals in which self and other views are severely hampered.

## Methods and materials

### Participants

Data were collected in the context of the “*Relations and Emotions in Parent-Adolescent Interaction Research*” (RE-PAIR) study, examining parent–adolescent interactions and adolescent depression in families with an adolescent with major depressive disorder (MDD)/dysthymia and families with an adolescent without psychopathology. Families were eligible for participation if the adolescent was aged 11–17 years, lived at home with at least one primary caregiver, at least one of the parents/caregivers was willing to participate in the study, and all had a good command of the Dutch language.

The current paper focuses on data from all parents (of both adolescents with MDD/dysthymia and adolescents without psychopathology) who participated in the fMRI part of the study. Eighty-five parents took part in this study. Six were excluded from data analyses for the following reasons: Brain abnormality (*n* = 1), ending the scan session due to symptoms of sleep apnea (*n* = 1), incomplete datasets due to technical issues (*n* = 3), and an a posteriori diagnosis in their adolescent child other than a primary diagnosis of MDD/dysthymia (*n* = 1).

The final sample consisted of 79 adults (mean [M]_age_ = 49.87 years, standard deviation [SD]_age_ = 4.62). Demographic and clinical characteristics of the sample are presented in Table 1. Based on the Mini International Neuropsychiatric Interview (M.I.N.I.; Sheehan et al., 1998), eight participants fulfilled criteria for a current psychiatric disorder: Obsessive-compulsive disorder (*n* = 1), dysthymia (*n* = 1), alcohol or drug dependency (*n* = 2), panic disorder (*n* = 1), generalized anxiety disorder (*n* = 3). Two participants fulfilled criteria for multiple current mental disorders, including mania, generalized anxiety disorder, and alcohol and drug dependency (*n* = 1) and depression, mania, and social phobia (*n* = 1). The mean CEM score in the sample was 17.97 (SD = 6.53). Fifteen participants reported moderate to extreme, 30 reported low-moderate, and 34 reported none or minimal levels of emotional abuse and/or emotional neglect.

**Table 1:** Demographic and clinical characteristics (*n* = 79)

Variables	Mean (SD) or n (%)	Range
Age, yr	49.87 (4.62)	40.5–60.9
Sex		
Females, *n* (%)	44 (55.7)	-
Males, *n* (%)	35 (44.3)	-
Race/ethnicity		
Multiracial, *n* (%)	1 (1.3)	-
White, *n* (%)	77 (97.4)	-
Other (Kurdish), *n* (%)	1 (1.3)	-
CEM (composite score EA and EN, CTQ-SF)	17.97 (6.53)	10–39
Emotional abuse (EA, CTQ-SF)	7.34 (3.04)	5–17
*None/minimal (5–8), n (%)*	59 (74.7)	-
*Low to moderate (9–12), n (%)*	14 (17.7)	-
*Moderate to extreme (≥13), n (%)*	6 (7.6)	-
Emotional neglect (EN, CTQ-SF)	10.63 (4.20)	5–23
*None/minimal (5–9), n (%)*	36 (45.6)	-
*Low to moderate (10–14), n (%)*	30 (38.0)	-
*Moderate to extreme (≥15), n (%)*	13 (16.4)	-
Physical abuse	5.38 (1.24)	5–14
*None/minimal (5–7), n (%)*	77 (97.4)	
*Low to moderate (8–9), n (%)*	1 (1.3)	
*Moderate to extreme (≥10), n (%)*	1 (1.3)	
Physical neglect	6.39 (1.67)	5–11
*None/minimal (5–7), n (%)*	66 (83.5)	
*Low to moderate (8–9), n (%)*	9 (11.4)	
*Moderate to extreme (≥10), n (%)*	4 (5.1)	
Sexual abuse	4.35 (1.37)	4–12
*None/minimal (5), n (%)*	75 (94.8)	
*Low to moderate (6–7), n (%)*	1 (1.3)	
*Moderate to extreme (≥8), n (%)*	3 (3.9)	
Anxiety severity (SCARED-A)	9.63 (7.89)	0–43
Depression severity (PHQ-9)	3.06 (3.97)	0–26
Self-esteem (RSES)	22.99 (5.51)	3–30
Right-handedness (EHI), *n* (%)	71 (89.9%)	-

*Notes.* SD, standard deviation; CEM, childhood emotional maltreatment; CTQ-SF, Childhood Trauma Questionnaire—Short Form (Arntz & Wessel, 1996); EA, emotional abuse; EHI, Edinburgh Handedness Inventory (Oldfield, 1971); EN, emotional neglect; PHQ-9, Patient Healthy Questionaire-9; RSES, Rosenberg Self-esteem Scale (Rosenberg); SCARED-A, Adult version of the Screen for Child Anxiety Related Emotional Disorders (Van Steensel & Bögels, 2014).

The study was approved by the medical ethical committee of the Leiden University Medical Centre (LUMC) (P17.241) and was conducted in accordance with the declaration of Helsinki and the Dutch Medical Research Involving Human Subjects Act (WMO). Details on the full study procedure can be found in Supplement 1. All hypotheses and analyses were preregistered (see https://osf.io/54nky). Part 1 of the preregistration focused on prolonged eye contact (i.e., direct vs. averted gaze) toward others (i.e., own child, unfamiliar child, and unfamiliar adult) and the self and has been published elsewhere (Wever et al., 2022). The current study relates to Part 2, which focused on prolonged eye contact and self-reported CEM and where we focus on the self and an unfamiliar other. Due to a multiplicity of findings and the contrast of self versus other being potentially affected by family and age factors (e.g., own child and unfamiliar child condition), we decided to focus on the contrast between self and an unfamiliar other adult in the main text. Nevertheless, we additionally analyzed all task conditions in a single 2 × 4 analysis of variance (ANOVA) model (as preregistered), including participants’ responses when viewing one’s own child or an unfamiliar child and self-reported CEM, of which the results are presented in Supplement 2.

#### Eye contact task

**Fig. 1:**
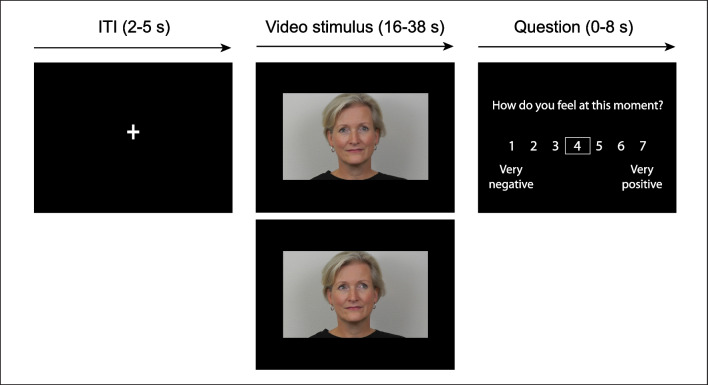
**Screens and timings of an unfamiliar other displaying direct gaze and averted gaze in the eye contact task. **The sex of the unfamiliar other target was matched with participants’ own sex

To characterize mood and neural responses to prolonged eye contact, participants performed the “eye contact” task (Wever et al. (2022), see Fig. 1 for an overview of the task). Participants were shown prerecorded videos of four targets: Themselves; an unfamiliar adult; their own child; and an unfamiliar child. Each video consisted of a single target looking straight into the camera (direct gaze) or averting their gaze to the left (averted gaze). We measured participants’ eye movements during the task using an eye-tracker. All videos were presented twice in two separate runs (2 × 4 × 2 = 16 trials in total). For the first run, all targets were presented in a random order. For each target, participants were presented with two successive videos of the same target, but with gaze direction randomly presented (i.e., starting with direct or averted gaze). For the second run, the order of targets was randomized again, but the order of the presentation of the gaze direction was counterbalanced to the first run. Video durations were based on randomly chosen intervals between 16–38 s from prerecorded videos of 45 s (based on Somerville et al., 2013). The first and last 3 s of each prerecorded video were discarded. Stimuli from each condition were presented for a total duration of 54 s across two runs, meaning that duration of a stimulus in a specific condition in run 2 was 54 s minus stimulus duration in run 1 with a minimum of 16 s. For this paper, we examined participants’ responses to direct versus averted gaze videos of themselves and a same-sex unfamiliar adult (e.g., unfamiliar other), which includes eight trials in total.

While in the scanner, participants were instructed to make eye contact with the targets in the videos. Each trial started with a fixation cross (duration 2–5 s), after which participants were presented with a video of themselves or an unfamiliar other looking directly into the camera or averting their gaze. After each video, participants reported on their mood (“How do you feel at this moment?”) on a Likert scale ranging from 1 (very negative) to 7 (very positive) and were instructed to answer and confirm the question within 8 s. If participants did not answer within the set time period of 8 s, the question duration included an extra 1 s during which a “Too late!” screen was shown. See Supplement 3 for information on stimulus development.

#### Childhood emotional maltreatment

The participants’ self-reported childhood maltreatment was assessed with the Dutch version of the Childhood Trauma Questionnaire Short Form before the scan session (CTQ-SF; Arntz & Wessel, 1996; Bernstein et al., 2003) and included the subscales: Emotional abuse (EN), emotional neglect (EN), physical abuse (PA), physical neglect (PN), and sexual abuse (SA). The present study focused on the subscales emotional abuse and emotional neglect of which the total scores were summed to create a composite score of childhood emotional maltreatment (CEM). This composite score could range from 10–50; higher scores indicated more experienced CEM. Item examples of the EA subscale are: “My family said hurtful or insulting things to me” and “I thought that my parents wished I had never been born.” Item examples of the EN subscale are: “I felt loved” and “My family felt close to each other.” The subscales, physical abuse, physical neglect, and sexual abuse, were used as control variables in this study to examine to what extent potential findings were unique to the context of CEM. All subscales consist of five items, except for the SA scale, which consisted of four items (item SA21 was removed, see Thombs et al., (2009)), and were answered on a Likert scale from 1 (never true) to 5 (very often true). The CTQ-SF is a sensitive and reliable questionnaire that has been validated in a Dutch sample (Thombs et al., 2009). Internal consistencies in the current sample were α = 0.79 for EA, α = 0.89 for EN, and α = 0.89 for the composite score. We log-transformed the CEM composite score and all individual subscales to account for skewness in the data.

#### Statistical analyses

Mood and gaze responses were analyzed in R (R Core Team (2013), version 3.6.1) with the following packages: Lme4 for mixed model analysis, psych for descriptive statistics, and ggplot2 for figures (Bates et al., 2012; Revelle, 2012; Wickham, Chang, & Wickham, 2016). Questions that were not answered within 8 s were reported as missing (*n* = 3/632, 0.5%) and were excluded from further analyses. Significance for analyses on mood and gaze responses was set at *p* < 0.05 (two-tailed) and Cohen’s *d* effect sizes were calculated for significant effects.

### Eye gaze acquisitions

Eye movements were recorded with a tower mounted monocular EyeLink 1000-Hz, MRI-compatible eye tracker (SR Research Ltd., Mississauga, Ontario, Canada), placed inside the scanner bore. We used a customized MATLAB (MathWorks, Inc., Natick, MA, version 9.5) script to preprocess raw eye movement data to calculate information on gaze position and duration. Using an established algorithm for face and facial feature detection (Viola & Jones, 2001), we created rectangular areas of interest (AOIs) around the left and right eye of the targets for all videos that were combined into a single AOI of the eye region for further analyses. The primary gaze measure was the percentage of dwell time within the eye region per video relative to the total video duration, in which dwell time is defined as the total amount of time spent looking within an AOI. Collection of gaze data of 31 participants was unsuccessful due to technical problems or a failed calibration procedure. Nine trials of four participants were excluded due to >30% missing gaze data per trial. This resulted in gaze data of 48 (out of 79) participants, including 375 trials (out of 384; 2.3% missing).

### fMRI data acquisition and analyses

MRI images were acquired by using a Philips 3.0T Achieva MRI scanner equipped with a SENSE-32 channel head coil. For the eye contact task, T2*-weighted echo planar imaging (EPI) was used and a structural 3D T1 scan was acquired (see Supplement 4 for details on scan parameters). MRI data were preprocessed and analyzed by using SPM12 (Wellcome Trust Centre for Neuroimaging, University College London). Functional MR images were slice-time corrected, corrected for field-strength inhomogeneities using b0 field maps, unwarped and realigned, co-registered to subject-specific structural images, normalized to MNI space using the DARTEL toolbox (Ashburner, 2007), and smoothed using an 8-mm, full-width, half-maximum isotropic Gaussian kernel. Raw and preprocessed data were checked for quality, registration, and movement. Average head movement did not exceed 1 voxel/3 mm for any of the participants (M = 0.09 mm, SD = 0.05 mm, range 0.002–2.76 mm). Furthermore, we corrected for serial autocorrelations by using a first-order autoregressive model (AR(1)). We removed low-frequency signals by using a high-pass filter (cutoff = 128 s) and included nuisance covariates to remove effects of run.

To examine participants’ neural responses to a direct gaze from self and an unfamiliar other (∆direct minus averted gaze contrast), we constructed a GLM with eight regressors indicating cue onset for each task condition (i.e., direct and averted gaze of own child, unfamiliar child, unfamiliar adult, self) and one regressor for subjective rating onsets. Cue onset regressors were defined from the onset of the video stimulus and modeled for the duration of this period (variable between 16–38 s). The subjective rating regressor was defined from the onset of each question and modeled for the duration the question was displayed on the screen (self-paced; *M*_duration_ = 3311 ms; SD_duration_ = 1316 ms; range 1029-9002 ms). We included six motion parameters (based on the realignment parameters) to correct for head motion. First, eight first-level SPM T-contrasts were specified for each task condition. T-contrast images of self and unfamiliar adult were entered in a 2 × 2 full factorial ANOVA design to examine task effects (for the 2 × 4 ANOVA design, including all task conditions, see Supplement 2). To examine associations between CEM and participants’ neural responses to looking either the self or another person in the eye (∆direct minus averted gaze contrast), we performed two separate whole-brain regression analyses with CEM scores as a between-subject regressor for videos of the self and an unfamiliar other separately. The first analysis tested for associations between interindividual variation in CEM scores and neural responses to gazing into one’s own eyes (i.e., ∆direct minus averted gaze in videos of the *self*). The second analysis tested for associations between interindividual variation in CEM scores and neural responses to gazing into someone else’s eyes (i.e., ∆direct minus averted gaze in videos of an unfamiliar other). All whole-brain results were corrected for multiple comparisons with family-wise error (FWE) cluster correction at *p* < 0.05 (with *p* < 0.001 cluster-forming threshold).

## Results

### Mood responses

**Fig. 2:**
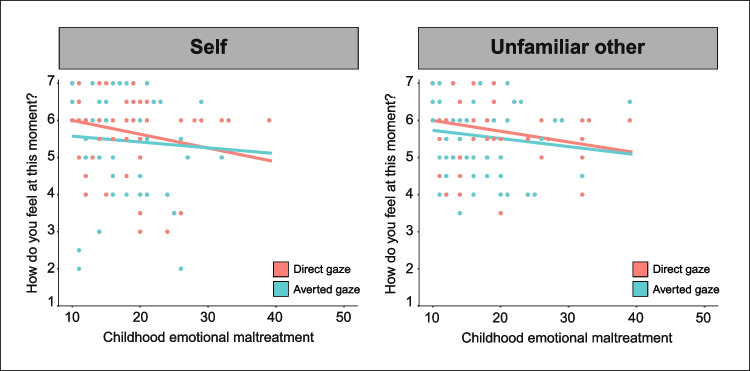
**Interaction between CEM and gaze direction (i.e., direct vs. averted gaze) on participants’ self-reported mood after the videos of self and unfamiliar other. **Participants with more CEM experiences reported a significantly lower mood after direct, but not averted gaze, compared with participants with fewer of such experiences (interaction CEM × gaze direction: *B* = 0.67, SE = 0.31, t(547) = 2.20, *p* = 0.028, *d* = 0.19). There was no significant interaction between CEM and target (i.e., self vs. other) (*p* = 0.970), nor between CEM, gaze direction, and target on participants’ self-reported mood responses (*p* = 0.191)

To examine whether CEM is associated with participants’ mood when gazing into one’s own or another person’s eyes, we performed a generalized linear mixed regression model with CEM, gaze direction (direct vs. averted), and target (self vs. unfamiliar other), and their interactions as predictors for participants’ mood responses. This analysis yielded a significant interaction between CEM and gaze direction (*B *= 0.67, SE = 0.31, t(547) = 2.20, *p* = 0.028, *d* = 0.19; Fig. 2). Post hoc analyses indicated that participants with higher levels of CEM reported a significantly less positive mood after direct gaze (*B* = −1.62, SE = 0.67, t(77) = −2.41, *p* = 0.018, *d* = 0.55) but not after averted gaze (*p* = 0.218). There was no significant interaction between CEM scores and target on participants’ mood responses (*p* = 0.970), indicating that participants’ mood did not differ after videos of themselves versus an unfamiliar other. There was no significant three-way interaction between CEM scores, target, and gaze direction on participants’ mood responses (*p* = 0.191). CEM was not significantly associated with participants’ self-reported mood, regardless of target or gaze direction (*p* = 0.068).

### Gaze responses

**Fig. 3:**
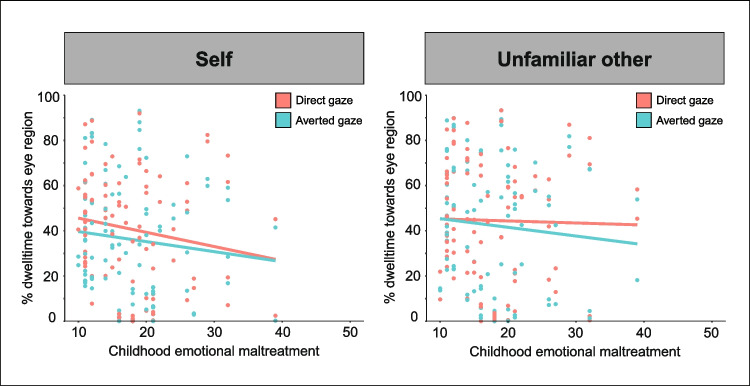
**Association between CEM and the percentage of dwell time within the eye region of targets relative to the total video duration for each gaze direction (i.e., direct vs. averted gaze) during videos of self and an unfamiliar other. **There was no significant interaction between CEM and gaze direction (*p* = 0.906) or CEM and target (*p* = 0.168) on the percentage of dwell time within the eye region of targets

To examine whether CEM is associated with the percentage of dwell time toward the eyes of the self and an unfamiliar other, we performed a generalized linear mixed regression model with CEM, gaze direction (direct vs. averted), and target (self vs. unfamiliar other), and their interactions as predictors for the percentage of dwell time within the eye region of the targets (Fig. 3). There were no significant interactions between CEM and target (*p* = 0.168) or CEM and gaze direction (*p* = 0.906), nor a significant three-way interaction between CEM, target, and gaze direction (*p* = 0.220) on dwell time within the eye region of the targets. Also, CEM was not associated with participants’ dwell time within eye region, regardless of target or gaze direction (*p* = 0.359).

### Neuroimaging results

**Fig. 4:**
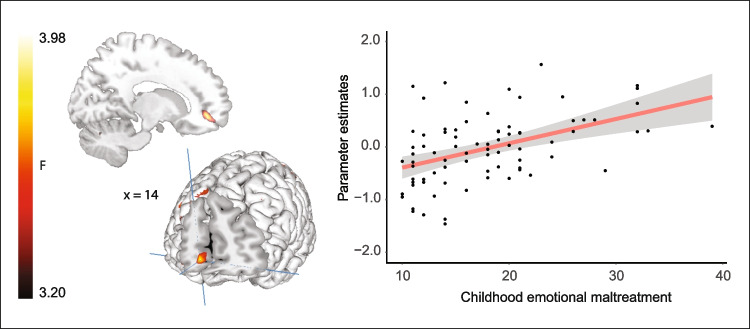
**CEM was associated with increased vmPFC activity when looking into one’s own eyes. **A whole-brain regression analysis testing for a positive association between CEM and neural responses to participants’ own direct gaze (Δ direct gaze – averted gaze) yielded a significant cluster in vmPFC (MNI-coordinate [14, 48, −8], *Z* = 3.98, *k* = 771, *p*_FWE-corr_ = 0.032 at cluster level). To visualize this association, we plotted parameter estimates in this region against self-reported CEM scores of participants. Regression lines are plotted for illustration purposes only. Whole-brain analyses were thresholded at *p* < 0.05 (FSW cluster-corrected using a cluster-forming threshold of *p* < 0.001)

To test for associations between CEM and neural responses to direct minus averted gaze of the self and an unfamiliar other, we performed two separate whole-brain regression analyses with participants’ experienced CEM scores as covariate of interest and their neural responses to direct gaze from either the self or unfamiliar other (Δdirect–averted gaze) as outcome variable. In response to participants’ own direct gaze, a significant positive association was found in the vmPFC (MNI-coordinate (14, 48, −8), *Z* = 3.98, *k* = 771, *p*_FWE-corr_ = 0.032 at cluster level), indicating that participants who reported more CEM showed enhanced BOLD-activation in vmPFC in response to their own direct versus averted gaze (Fig. 4). In response to the gaze of an unfamiliar other person, we found no significant association between CEM and neural responses to direct (vs. averted gaze). See Supplement 5 for main effect of target on participants’ neural responses during the task at whole-brain level. There was no main effect of gaze direction at the neural level.

### Covariate analyses

Given the associations between CEM and self-esteem (*r* = −0.37, *p* < 0.001) and symptom severity of anxiety (*r* = 0.34,* p* = 0.002) and depression (*r* = 0.23, *p* = 0.043) in the current sample (Supplement 6), we ran a set of analyses to elucidate whether these measures might mediate the reported associations. All analyses were controlled for participants’ sex, and neuroimaging analyses were additionally controlled for handedness (left/right) and psychotropic medication status (yes/no) (see Supplement 7 for detailed information about these measures).

Associations between CEM and participants’ mood responses remained significant after controlling for participants’ sex, anxiety or depression symptom severity, and self-esteem. The association between CEM and enhanced vmPFC activation in response to participants’ own direct gaze remained significant after controlling for sex and handedness but was no longer significant when separately controlling for psychotropic medication status, severity of anxiety or depression symptoms, or self-esteem levels. We used a method to examine the sequential contribution of each of the regressors and averaged them over all possible sequential orderings to calculate the relative importance of each regressor (Relaimpo package in R). This analysis showed that childhood emotional maltreatment explained most of the variance (82.12%) of the relationship between childhood emotional maltreatment levels and enhanced vmPFC activation. Self-esteem explained 10.49%, anxiety severity explained 4.72%, and depression severity explained 2.67%. Together this shows that variance in vmPFC to one’s own gaze is mostly driven by individual differences in childhood emotional maltreatment and to a lesser extent by self-esteem, depression, and anxiety.

To further explore whether our results could be explained by other types of childhood maltreatment (i.e., physical abuse, physical neglect, and sexual abuse), we ran non-preregistered analyses in which we included these types of childhood maltreatment as covariates in the generalized linear mixed regression models when testing for the associations between CEM and 1) mood and 2) gaze behavior. All reported relationships between CEM and mood and gaze behavior as reported above remained intact. Furthermore, we controlled for physical abuse, physical neglect, and sexual abuse in the whole-brain regression analyses between CEM and neural responses to gazing into one’s own and another person’s eyes. The association between vmPFC activation and childhood emotional maltreatment remained significant after adding physical abuse and sexual abuse to the model, suggesting that this effect could not be explained by these types of childhood maltreatment. However, adding physical neglect to the regression model did not result in clusters that survived correction for multiple comparisons. This might suggest that physical neglect is, at least partly, associated with the enhanced vmPFC activation when participants are gazing into their own eyes in the task. Conversely, considering that the severity of childhood maltreatment in this sample was mostly composed of emotional abuse, emotional neglect, and physical neglect, and to a lesser extent of physical abuse and sexual abuse, it might suggest a general effect of childhood maltreatment. Because this cannot be disentangled based on the data of the current study, it is of interest to focus on this matter in a larger sample that includes a more balanced prevalence of all types of childhood maltreatment.

### Exploratory analyses

In addition, we explored whether participants’ self-reported mood, gaze, and neural responses were uniquely associated with emotional abuse and emotional neglect. See Supplement 8 for a detailed overview of the results of these exploratory analyses. It should be noted that these analyses were not preregistered and thus exploratory in nature. Our sample size was not a priori determined to have sufficient power to detect these nuanced effects. The results of these analyses need to be interpreted in the light of these limitations.

## Discussion

This study shows that people who report moderate-to-extreme levels of CEM show enhanced vmPFC responses to one’s own direct gaze but not to the gaze of a stranger. CEM’s effects on mood generalized to eye contact with other people, showing that CEM was not only associated with lower mood after directly gazing into one’s own eyes but also after gazing into another person’s eyes. CEM was not associated with participants’ gaze responses into one’s own eyes or the eyes of a stranger in neither of the gaze directions.

Increased vmPFC activity in response to participants’ own direct gaze fits into a larger literature robustly linking vmPFC activity to affective aspects of self-evaluation (Moran et al., 2006; Northoff et al., 2006; Talmon et al., 2021; van der Meer et al., 2010; Will et al., 2020; Will et al., 2017). Given the vmPFCs general role in emotion-regulation and representing subjective value, increased vmPFC activity in participants reporting CEM might indicate an increased engagement of self-related, emotion-regulation processes in response to their own direct gaze. As such, vmPFC hyperactivation might signal a potential neural phenotype related to (maladaptive) patterns of increased self-referential processing associated with CEM that is activated when being confronted with one’s own gaze. This is in line with a study by Talmon et al. (2021), who found enhanced default mode network activation, including the same vmPFC region, in people diagnosed with social anxiety disorder and who reported to have a history of CEM. Interestingly, they did not find this effect in people diagnosed with social anxiety disorder without a history of CEM. Hyperactivation in a similar area of the vmPFC [MNI-coordinate 10, 44, −7] also was found in depressed patients (vs. healthy controls) in response to judgements about whether personality traits described themselves (Yoshimura et al., 2010), in particular for negative traits. The overlap between the clusters found in patients with social anxiety disorder and depression, and the cluster covarying with CEM in our sample, suggests that this region may be involved in negative self-attributions, either explicitly or in a more implicit manner (i.e., when looking at the self). The associations between CEM and vmPFC activation did not remain significant when controlling for participants’ depression and anxiety severity, psychotropic medication status, or self-esteem. As indicated by their individual correlations, these variables show an association with CEM but are not directly related to vmPFC activation. Therefore, CEM seems to be uniquely associated with participants’ neural response to one’s own direct gaze.

While people reporting less or no CEM showed a higher mood in response to direct versus averted gaze, this was not the case for individuals reporting more CEM, suggesting that people who with CEM do not benefit as much from the mood-boosting effects of making eye contact as people who reported less or no CEM. We found no evidence of CEM specifically moderating mood responses to videos of the self versus others. This is in contrast with participants’ neural responses, which showed a unique association of CEM on self-related, but not other-related, stimuli in our sample. Although the reason for this discrepancy is not entirely clear, it suggests that higher CEM levels do not result in differential mood responses when seeing oneself versus a stranger.

CEM did not affect people’s amount of gaze into one’s own or other’s eyes. Nor did CEM differentially affect how much people gazed into the eyes of the targets during direct versus averted gaze videos. This is not in line with our hypotheses and clinical observations that people reporting more CEM show a greater tendency to avoid eye contact (Krill, 2011; Wilkinson, 2010). A possible explanation for this discrepancy is that the stimuli used in the eye contact task were prerecorded videos instead of real-life mutual gaze encounters similar to the clinical observations. This is emphasized by studies showing that eye contact only elevated participants’ levels of arousal in case of real-life bidirectional eye contact (Hietanen et al., 2020; Jarick & Bencic, 2019). Future studies on eye contact during real-life interactions should elucidate whether people’s gaze toward one’s own or others’ eyes covaries with people’s reported CEM levels while receiving real-time visual feedback from the eyes of oneself (e.g., via a mirror) and others.

Observed associations between CEM and participants’ neural responses is striking given that the average age of our current sample was ±50 years, suggesting that the impact of CEM is still discernable well into adulthood. Moreover, our sample was not recruited based on participants having extreme levels of CEM. Thus, reported associations between brain and mood with CEM cannot be explained by a selection bias and seem to be even present in people who experienced moderate levels of CEM. In addition to these strengths, our study has limitations. CEM was retrospectively reported by the participants about their childhood, which makes it susceptible to response biases. However, studies suggest that underreporting CEM is more common than overreporting (Maughan & Rutter, 1997), indicating that participants’ CEM levels in the current study are probably representative for, or on the lower end of, the level of CEM to which they were exposed. Although results match well-established findings reporting that people who experienced CEM might be more self-conscious and have enhanced negative self-attributions (Alloy et al., 2006; Gibb, 2002; Talmon et al., 2021; van Harmelen et al., 2010), we did not ask participants to report their self-views (e.g., self-disgust, self-compassion) in response to their own direct and averted gaze. Therefore, we cannot pinpoint which psychological processes may have driven the increased neural reactivity in vmPFC. Future studies might benefit from assessing self-cognitions to examine how they mediate people’s responses to their own gaze. Because of the technical challenges of measuring eye gaze in the scanner, we successfully collected gaze data from 48 of the 79 participants; therefore, the results regarding participants gaze responses should be interpreted in the light of this reduced sample size. Lastly, given that previous studies have shown unique effects of emotional abuse and emotional neglect on individuals social and emotional functioning (Milojevich et al., 2019; Warmingham et al., 2019), it is of interest to examine these unique (neural) correlates of the impact of emotional abuse and neglect on participants’ responses to eye contact in a larger sample.

Our results show that childhood emotional maltreatment is associated with enhanced neural responses to one’s own gaze. This impact on self-related processing seemed more pronounced for automatic processes (i.e., neural responses) and may be associated with more spontaneous affective reactions in people reporting more emotional maltreatment when gazing into one’s own eyes (e.g., sadness or self-loathing). Future studies should investigate whether treatments that focus on the strengthening of participants’ self-views (e.g., mindfulness-based cognitive therapy focused on negative self-referential processing or self-esteem training (Korrelboom et al., 2012; Lovas & Schuman-Olivier, 2018)) are beneficial for people who experienced CEM and whether such therapeutic interventions are able to normalize their behavioral and neural responses when connecting with oneself.

### Supplementary Information

Below is the link to the electronic supplementary material.

Supplementary file1 (DOCX 791 KB)

## Data Availability

The de-identified data, analysis scripts and materials for this study are available on DataverseNL and the MRI data are available on NeuroVault (https://neurovault.org/collections/UMDSFUUL/). All study measures, hypotheses, and analyses were preregistered at Open Science Framework before data analyses (https://osf.io/54nky). For any questions or additional material, please contact the corresponding author.
