# Supplementary material for: Looking into troubled waters: Childhood emotional maltreatment modulates neural responses to prolonged gazing into one’s own, but not others’, eyes
Source: Cogn Affect Behav Neurosci. 2023 Oct 25;23(6):1598–609. doi: 10.3758/s13415-023-01135-y (PMC10684401; doi:10.3758/s13415-023-01135-y)
Supplement: Supplementary file 1 — Supplementary file1 (DOCX 791 KB) [file 13415_2023_1135_MOESM1_ESM.docx]

**Looking into Troubled Waters: Childhood Emotional Maltreatment modulates Neural Responses to Prolonged Gazing into One’s Own, but not Others’, Eyes.**

Mirjam C.M. Wever^a,b^, Lisanne A.E.M. van Houtum^a,b^, Loes H.C. Janssen^a,b^, Wilma G.M. Wentholt^a,b^, Iris M. Spruit^a^, Marieke S. Tollenaar^a,b^, Geert-Jan Will^c^, Bernet M. Elzinga^a,b^.

^a^ Department of Clinical Psychology, Leiden University, Leiden, the Netherlands

^b^ Leiden Institute for Brain and Cognition, Leiden, the Netherlands

^c^ Department of Clinical Psychology, Utrecht University, Utrecht, the Netherlands

Corresponding author

Mirjam C.M. Wever, Leiden University, Faculty of Social and Behavioral Sciences, 2300 RB Leiden, The Netherlands. E-mail: m.c.m.wever@fsw.leidenuniv.nl

Co-author details

M.C.M. Wever, m.c.m.wever@fsw.leidenuniv.nl

Lisanne A.E.M. van Houtum, [l.a.e.m.van.houtum@fsw.leidenuniv.nl](mailto:l.a.e.m.van.houtum@fsw.leidenuniv.nl)

Loes H.C. Janssen, [l.h.c.janssen@fsw.leidenuniv.nl](mailto:l.h.c.janssen@fsw.leidenuniv.nl)

Wilma G.M. Wentholt, [w.g.m.wentholt@fsw.leidenuniv.nl](mailto:w.g.m.wentholt@fsw.leidenuniv.nl)

Iris M. Spruit, [i.m.spruit@fsw.leidenuniv.nl](mailto:i.m.spruit@fsw.leidenuniv.nl)

Dr. Marieke S. Tollenaar, [m.s.tollenaar@fsw.leidenuniv.nl](mailto:m.s.tollenaar@fsw.leidenuniv.nl)

Dr. Geert-Jan Will, [g.j.will@uu.nl](mailto:g.j.will@uu.nl)

**Abstract**

One of the most prevalent nonverbal, social phenomena known to automatically elicit self- and other-referential processes is eye contact. By its negative effects on the perception of social safety and views about the self and others, childhood emotional maltreatment (CEM) may fundamentally affect these processes. To investigate whether the socio-affective consequences of CEM may become visible in response to (prolonged) eye gaze, 79 adult participants (M*_age_* = 49.87, SD *_age_* = 4.62) viewed videos with direct and averted gaze of an unfamiliar other or themselves while we recorded self-reported mood, eye movements using eye-tracking, and markers of neural activity using fMRI. Participants who reported higher levels of CEM exhibited increased activity in ventromedial prefrontal cortex to one’s own, but not to others’, direct gaze. Furthermore, in contrast to those who reported fewer of such experiences, they did not report a better mood in response to a direct gaze of self and others, despite equivalent amounts of time spent looking into their own and other peoples’ eyes. The fact that CEM is associated with enhanced neural activation in a brain area that is crucially involved in self-referential processing (i.e., vmPFC) in response to one’s own direct gaze is in line with the chronic negative impact of CEM on a person’s self-views. Interventions that directly focus on targeting maladaptive self-views elicited during eye gaze to self may be clinically useful.

**Keywords:** Childhood Emotional Maltreatment, Self-referential Processing, Direct Gaze, Ventromedial Prefrontal Cortex, Eye Tracking, Social Cognition

**Introduction**

Childhood emotional maltreatment (CEM) is common with global prevalence rates between 18.4-36.3% (Stoltenborgh, Bakermans‐Kranenburg, Alink, & van IJzendoorn, 2015). It encompasses situations in which parents fail to accurately perceive and appropriately respond to the emotional and psychological needs of their child (Egeland, 2009; Gilbert et al., 2009). CEM is defined as any act of commission (i.e., emotional abuse) or omission (i.e., emotional neglect) of behavior that is (potentially) harmful or insensitive to the child’s emotional development. It is considered one of the most devastating forms of maltreatment due to its chronic exposure pattern from an early age onwards and the involvement of a primary caregiver.

CEM gives rise to long-term negative consequences into adulthood (Egeland, 2009; Gilbert et al., 2009; Reyome, 2010; Spertus, Yehuda, Wong, Halligan, & Seremetis, 2003; van Harmelen et al., 2010). One of these potential outcomes is the development of negative cognitions about the self and others as a result of the perceived betrayal of trust during childhood by a primary caregiver (Baugh, Cox, Young, & Kealy, 2019). On the one hand CEM may result in the generalization of distrust to others leading to maladaptive other-schemas (e.g., everyone has bad intentions), while on the other hand it may also fuel maladaptive schemas about the self leading to the believe that they are unloved, worthless, or unwanted (Baugh et al., 2019; Gobin & Freyd, 2014). Although studies including a direct comparison between responses to the self and others are sparse, people with a history of CEM seems to be particularly vulnerable to develop negative self-views when compared to other types of maltreatment (i.e., physical or sexual abuse) (Alloy, Abramson, Smith, Gibb, & Neeren, 2006; Gibb, 2002; Gibb, Abramson, & Alloy, 2004; Rose & Abramson, 1992; van Harmelen et al., 2010). In turn, negative self-views put people with a history of CEM at a greater risk for developing internalizing disorders, such as anxiety disorders and depression, and can contribute to interpersonal difficulties and problems in the formation and maintenance of (intimate) relationships (Reyome, 2010; Riggs, 2010; Wright, Crawford, & Del Castillo, 2009).

One of the most prevalent nonverbal, social phenomena known to automatically elicit both self- and other-referential processes is eye contact (Conty, George, & Hietanen, 2016). Eye contact with others generally elicits positive feelings (Hietanen, 2018; Wever et al., 2022). However, people who were abused as a child often perceive eye contact as a signal of threat (Krill, 2011; Wilkinson, 2010). Hence, avoiding eye contact may be an automatic response in individuals with CEM as a means to cope with negative affect caused by face-to-face interactions (Tottenham et al., 2011). In addition to *social* eye contact, eye contact with oneself (e.g., in the mirror) elicit strong emotional responses related to the self in people with low self-esteem, including feelings of shame and disgust (Erdem, 2019; Ypsilanti, Robson, Lazuras, Powell, & Overton, 2020). However, how a history of CEM may affect the processing of eye contact with oneself has yet to be determined.

While there is a dearth of studies on neural circuitry supporting self- and other-referential processes during prolonged eye gazing to the self and others, neuroimaging studies have identified a consistent network of regions that responds to static pictures or judgements of the self and others, consisting of distinct parts of the medial prefrontal cortex, insula, temporoparietal junction (TPJ), posterior cingulate cortex (PCC), and cuneus (D'Argembeau, 2013; Denny, Kober, Wager, & Ochsner, 2012; Lemogne et al., 2011; Murray, Schaer, & Debbané, 2012; Northoff et al., 2006; van der Meer, Costafreda, Aleman, & David, 2010). Especially the ventromedial prefrontal cortex (vmPFC) and dorsomedial prefrontal cortex (dmPFC) seem to lay at opposite ends of a functional spectrum representing the processing of affective self-relevant and other-relevant information, respectively (Denny et al., 2012).

Literature on the association between CEM and neural responses to self- and other-related content is limited to the finding that people with a history of CEM exhibited enhanced amygdala reactivity in response to neutral and emotional faces (Dannlowski et al., 2012; Tottenham et al., 2011; van Harmelen et al., 2013). Only a few studies have examined whether a history of CEM is associated with neural responses to self-related content (Puetz et al., 2021; Talmon, Dixon, Goldin, Heimberg, & Gross, 2021), but none of them examined the relationship between a history of CEM and gazing into one’s own and others’ eyes. This is striking, since eye contact with others is fundamental to our daily lives and plays an important role in our social connections with others (Emery, 2000). In addition, connecting with ourselves (e.g., via a mirror) elicits powerful affective and physiological responses, which facilitates identification and mitigation of one’s (maladaptive) responses when being confronted with oneself (Baldwin, 1996; Vergallito et al., 2020).

To better understand processes supporting eye contact with the self and others in individuals with a history of CEM, we examined associations between self-reported CEM and participants’ mood, gaze, and neural responses to direct and averted gaze of themselves and an unfamiliar other adult. First, we expected that people reporting higher levels of CEM would report lower mood after direct (vs averted) gaze videos, and that they would gaze less often into the eyes of self and others compared to people who report lower levels of CEM. Given that there are no studies examining the association between experienced CEM and people’s neural responses to gazing into one’s own and others’ eyes, our neuroimaging analyses are exploratory. Examining how individuals with a history of CEM respond to their own or other people’s direct gaze might not only yield new insights in fundamental processes of human nature, but may also contribute to new interventions for individuals in which self and other views are severely hampered.

**Methods and Materials**

***Participants***

Data were collected in the context of the “Relations and Emotions in Parent-Adolescent Interaction Research” (RE-PAIR) study, examining parent-adolescent interactions and adolescent depression in families with an adolescent with Major Depressive disorder (MDD)/dysthymia and families with an adolescent without psychopathology. Families were eligible for participation in case the adolescent was aged between 11-17 years, lived at home with at least one primary caregiver, at least one of the parents/caregivers was willing to participate in the study, and all had a good command of the Dutch language.

The current paper focuses on data from all parents (of both adolescents with MDD/dysthymia and adolescents without psychopathology) who participated in the fMRI part of the study. Eighty-five parents took part in this study and six were excluded from data analyses due to a brain abnormality (*n*=1), ending the scan session due to symptoms of sleep apnea (*n*=1), incomplete datasets due to technical issues (*n*=3), and an a posteriori diagnosis in their adolescent child other than a primary diagnosis of MDD/dysthymia (*n*=1).

The final sample consisted of 79 adults (*M*_age_=49.87 years, SD_age_=4.62). Demographic and clinical characteristics of the sample are presented in Table 1. Based on the Mini International Neuropsychiatric Interview (M.I.N.I.; (Sheehan et al., 1998)), eight participants fulfilled criteria for a current psychiatric disorder, i.e., obsessive-compulsive disorder (*n*=1), dysthymia (*n*=1), alcohol or drugs dependency (*n*=2), panic disorder (*n*=1), generalized anxiety disorder (*n*=3). Two participants fulfilled criteria for multiple current mental disorders, including mania, generalized anxiety disorder, and alcohol- and drugs dependency (*n*=1) and depression, mania, and social phobia (*n*=1). The mean CEM score in the sample was 17.97 (SD=6.53). *n*=15 participants reported moderate to extreme, *n*=30 low-moderate, and *n*=34 none or minimal levels of emotional abuse and/or emotional neglect.

The study was approved by the medical ethical committee of the Leiden University Medical Centre (LUMC) (P17.241) and was conducted in accordance with the declaration of Helsinki and the Dutch Medical Research Involving Human Subjects Act (WMO). Details on the full study procedure can be found in Supplement 1. All hypotheses and analyses were preregistered (see [https://osf.io/54nky](https://osf.io/54nky/?view_only=b55315eee49348e0833ced3746ae763f)). Part 1 of the preregistration focused on prolonged eye contact (i.e., direct versus averted gaze) towards others (i.e., own child, unfamiliar child and unfamiliar adult) and the self, and has been published elsewhere (Wever et al., 2022). The current study relates to Part 2, which focuses on prolonged eye contact and self-reported CEM and where we focus on the self and an unfamiliar other. Due to a multiplicity of findings and the contrast of self versus other being potentially affected by family and age factors (e.g., own child and unfamiliar child condition), we decided to focus on the contrast between self and an unfamiliar other adult in the main text. Nevertheless, we additionally analyzed all task conditions in a single 2 × 4 ANOVA model (as preregistered), including participants’ responses when viewing one’s own child or an unfamiliar child and self-reported CEM, of which the results are presented in Supplement 2.

**Table 1.** Demographic and clinical characteristics (*n*=79)

| Variables | Mean (SD) or n (%) | Range |
| --- | --- | --- |
| Age, years | 49.87 (4.62) | 40.5 – 60.9 |
| Sex |  |  |
| Females, *n* (%) | 44 (55.7) | - |
| Males, *n* (%) | 35 (44.3) | - |
| Race/Ethnicity |  |  |
| Multiracial, *n* (%) | 1 (1.3) | - |
| White, *n* (%) | 77 (97.4) | - |
| Other (Kurdish), *n* (%) | 1 (1.3) | - |
| CEM (composite score EA and EN, CTQ-SF) | 17.97 (6.53) | 10 – 39 |
| Emotional abuse (EA, CTQ-SF) | 7.34 (3.04) | 5 – 17 |
| *None/minimal (5-8)*, *n* (%) | 59 (74.7) | - |
| *Low to moderate (9-12)*, *n* (%) | 14 (17.7) | - |
| *Moderate to extreme (≥13)*, *n* (%) | 6 (7.6) | - |
| Emotional neglect (EN, CTQ-SF) | 10.63 (4.20) | 5 – 23 |
| *None/minimal (5-9)*, *n* (%) | 36 (45.6) | - |
| *Low to moderate (10-14)*, *n* (%) | 30 (38.0) | - |
| *Moderate to extreme (≥15)*, *n* (%) | 13 (16.4) | - |
| Physical abuse | 5.38 (1.24) | 5 – 14 |
| *None/minimal (5-7)*, *n* (%) | 77 (97.4) |  |
| *Low to moderate (8-9)*, *n* (%) | 1 (1.3) |  |
| *Moderate to extreme (≥10)*, *n* (%) | 1 (1.3) |  |
| Physical neglect | 6.39 (1.67) | 5 – 11 |
| *None/minimal (5-7)*, *n* (%) | 66 (83.5) |  |
| *Low to moderate (8-9)*, *n* (%) | 9 (11.4) |  |
| *Moderate to extreme (≥10)*, *n* (%) | 4 (5.1) |  |
| Sexual abuse | 4.35 (1.37) | 4 – 12 |
| *None/minimal (5)*, *n* (%) | 75 (94.8) |  |
| *Low to moderate (6-7)*, *n* (%) | 1 (1.3) |  |
| *Moderate to extreme (≥8)*, *n* (%) | 3 (3.9) |  |
| Anxiety severity (SCARED-A) | 9.63 (7.89) | 0 – 43 |
| Depression severity (PHQ-9) | 3.06 (3.97) | 0 – 26 |
| Self-esteem (RSES) | 22.99 (5.51) | 3 – 30 |
| Right-handedness (EHI), *n* (%) | 71 (89.9%) | - |

*Notes.* SD, Standard deviation; CEM, Childhood Emotional Maltreatment; CTQ-SF, Childhood Trauma Questionnaire – Short Form (Arntz & Wessel, 1996); EA, Emotional abuse; EHI, Edinburgh Handedness Inventory (Oldfield, 1971); EN, Emotional neglect; PHQ-9, Patient Healthy Questionaire-9; RSES, Rosenberg Self-esteem Scale (Rosenberg); SCARED-A, Adult version of the Screen for Child Anxiety Related Emotional Disorders (Van Steensel & Bögels, 2014).

***Eye contact task***

To characterize mood and neural responses to prolonged eye contact, participants performed the “eye contact” task (Wever et al. (2022), see Figure 1 for an overview of the task). Participants were shown pre-recorded videos of four targets: Themselves, an unfamiliar adult, their own child and an unfamiliar child. Each video consisted of a single target looking straight into the camera (direct gaze) or averting their gaze to the left (averted gaze). We measured participants’ eye movements during the task using an eye-tracker. All videos were presented twice in two separate runs (2 × 4 × 2 = 16 trials in total). For the first run, all targets were presented in a random order. For each target, participants were presented with two successive videos of the same target, but with gaze direction randomly presented (i.e., starting with direct or averted gaze). For the second run, the order of targets was randomized again, but the order of the presentation of the gaze direction was counterbalanced to the first run. Video durations were based on randomly chosen intervals between 16-38s from prerecorded videos of 45s (based on (Somerville et al., 2013)). The first and last 3s of each prerecorded video were discarded. Stimuli from each condition were presented for a total duration of 54s across two runs, meaning that duration of a stimulus in a specific condition in run 2 was 54s minus stimulus duration in run 1 with a minimum of 16s. For this paper, we examined participants’ responses to direct versus averted gaze videos of themselves and a same-sex unfamiliar adult (e.g., unfamiliar other), which includes 8 trials in total.

While in the scanner, participants were instructed to make eye contact with the targets in the videos. Each trial started with a fixation cross (duration: 2-5s), after which participants were presented with a video of themselves or an unfamiliar other looking directly into the camera or averting their gaze. After each video, participants reported on their mood (“How do you feel at this moment?”) on a Likert scale ranging from 1 (very negative) to 7 (very positive) and were instructed to answer and confirm the question within 8s. In case participants did not answer within the set time period of 8s, the question duration included an extra 1s during which a “Too late!” screen was shown. See Supplement 3 for information on stimulus development.


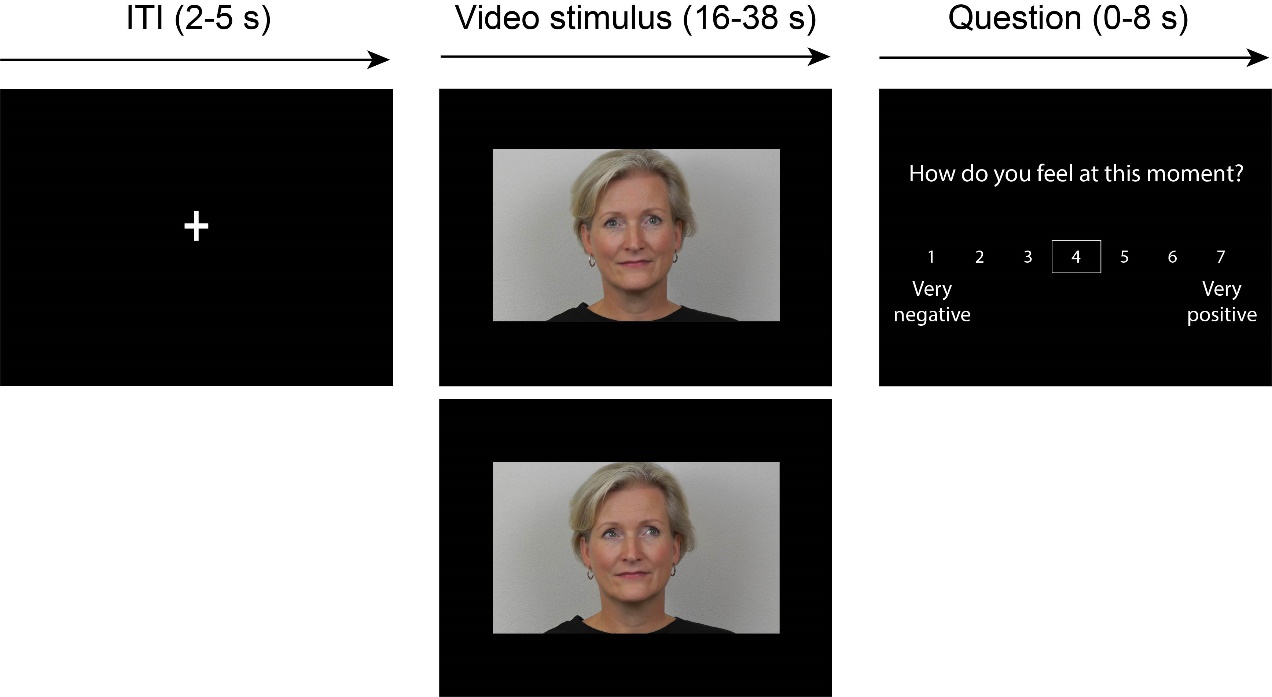


**Figure 1.** **Screens and timings of an unfamiliar other displaying direct gaze and averted gaze in the eye contact task.** The sex of the unfamiliar other target was matched with participants’ own sex.

***Childhood emotional maltreatment***

Participants’ self-reported childhood maltreatment was assessed with the Dutch version of the Childhood Trauma Questionnaire Short Form prior to the scan session (CTQ-SF; (Arntz & Wessel, 1996; Bernstein et al., 2003)), and included the subscales emotional abuse (EN), emotional neglect (EN), physical abuse (PA), physical neglect (PN), and sexual abuse (SA). The present study focused on the subscales emotional abuse and emotional neglect, of which the total scores were summed to create a composite score of childhood emotional maltreatment (CEM). This composite score could range between 10-50, with higher scores indicating more experienced CEM. Item examples of the EA subscale are: “My family said hurtful or insulting things to me” and “I thought that my parents wished I had never been born”. Item examples of the EN subscale are: “I felt loved” and “My family felt close to each other”. The subscales physical abuse, physical neglect, and sexual abuse were used as control variables in this study in order to examine to what extent potential findings were unique to the context of CEM. All subscales consist of five items, except for the SA scale which consisted of four items (item SA21 was removed, see Thombs, Bernstein, Lobbestael, and Arntz (2009)), and were answered on a Likert scale from 1 (never true) to 5 (very often true). The CTQ-SF is a sensitive and reliable questionnaire and has also been validated in a Dutch sample (Thombs et al., 2009). Internal consistencies in the current sample were α = 0.79 for EA, α = 0.89 for EN, and α = 0.89 for the composite score. We log-transformed the CEM composite score and all individual subscales to account for skewness in the data.

***Statistical analyses***

Mood and gaze responses were analyzed in R (R Core Team (2013), version 3.6.1) with the following packages: Lme4 for mixed model analysis, psych for descriptive statistics and ggplot2 for figures (Bates et al., 2012; Revelle, 2012; Wickham, Chang, & Wickham, 2016). Questions that were not answered within 8s were reported as missing (n=3/632, 0.5%) and were excluded from further analyses. Significance for analyses on mood and gaze responses was set at *p*<0.05 (two-tailed) and Cohen’s *d* effect sizes were calculated for significant effects.

*Eye gaze acquisitions*

Eye movements were recorded with a tower mounted monocular EyeLink 1000 Hz MRI-compatible eye tracker (SR Research Ltd., Mississauga, Ontario, Canada), placed inside the scanner bore. We used a customized MATLAB (MathWorks, Inc., Natick, MA, version 9.5) script to preprocess raw eye movement data to calculate information on gaze position and duration. Using an established algorithm for face and facial feature detection (Viola & Jones, 2001) we created rectangular areas of interest (AOIs) around the left and right eye of the targets for all videos that were combined into a single AOI of the eye region for further analyses. The primary gaze measure was the percentage of dwell time within the eye region per video relative to the total video duration, in which dwell time is defined as the total amount of time spent looking within an AOI. Collection of gaze data of 31 participants was unsuccessful due to technical problems or a failed calibration procedure. Nine trials of four participants were excluded due to >30% missing gaze data per trial. This resulted in gaze data of 48 (out of 79) participants, including 375 trials (out of 384; 2.3% missing).

*fMRI Data Acquisition and analyses*

MRI images were acquired using a Philips 3.0T Achieva MRI scanner equipped with a SENSE-32 channel head coil. For the eye contact task, T2*-weighted echo planar imaging (EPI) was used and a structural 3D T1 scan was acquired (see Supplement 4 for details on scan parameters). MRI data were preprocessed and analyzed using SPM12 (Wellcome Trust Centre for Neuroimaging, University College London). Functional MR images were slice-time corrected, corrected for field-strength inhomogeneities using b0 field maps, unwarped and realigned, co-registered to subject-specific structural images, normalized to MNI space using the DARTEL toolbox (Ashburner, 2007), and smoothed using an 8-mm full width half maximum isotropic Gaussian kernel. Raw and preprocessed data were checked for quality, registration, and movement. Average head movement did not exceed 1 voxel/3mm for any of the participants (*M*=0.09mm, SD=0.05mm, range: 0.002–2.76mm). Furthermore, we corrected for serial autocorrelations using a first order autoregressive model (AR(1)). We removed low-frequency signals using a high-pass filter (cutoff=128s) and included nuisance covariates to remove effects of run.

To examine participants’ neural responses to a direct gaze from self and an unfamiliar other (∆direct minus averted gaze contrast) we constructed a GLM with eight regressors indicating cue onset for each task condition (i.e., direct and averted gaze of own child, unfamiliar child, unfamiliar adult, self) and one regressor for subjective rating onsets. Cue onset regressors were defined from the onset of the video stimulus and modeled for the duration of this period (variable between 16-38s). The subjective rating regressor was defined from the onset of each question and modeled for the duration the question was displayed on the screen (self-paced; *M*_duration_=3311ms; SD_duration_=1316ms; range: 1029-9002ms). We included 6 motion parameters (based on the realignment parameters) to correct for head motion. First, eight first-level SPM T-contrasts were specified for each task condition. T-contrast images of self and unfamiliar adult were entered in a 2 × 2 full factorial ANOVA design to examine task effects (for the 2 × 4 ANOVA design including all task conditions, see Supplement 2). To examine associations between CEM and participants’ neural responses to looking either the self or another person in the eye (∆direct minus averted gaze contrast) , we performed two separate whole-brain regression analyses with CEM scores as a between-subject regressor for videos of the self and an unfamiliar other separately. The first analysis tested for associations between inter-individual variation in CEM scores and neural responses to gazing into one’s own eyes (i.e., (∆direct minus averted gaze in videos of the *self)*. The second analysis tested for associations between inter-individual variation in CEM scores and neural responses to gazing into someone else’s eyes (i.e., (∆direct minus averted gaze in videos of an unfamiliar other). All whole-brain results were corrected for multiple comparisons with Family-Wise Error (FWE) cluster correction at *p*<0.05 (with a *p*<0.001 cluster-forming threshold).

**Results**

*Mood responses*

To examine whether CEM is associated with participants’ mood when gazing into one’s own or another person’s eyes, we performed a generalized linear mixed regression model with CEM, gaze direction (direct vs averted), and target (self vs unfamiliar other), and their interactions as predictors for participants’ mood responses. This analysis yielded a significant interaction between CEM and gaze direction (*B*=0.67, SE=0.31, t(547)=2.20, *p*=0.028, *d*=0.19; Figure 2). Post-hoc analyses indicated that participants with higher levels of CEM reported a significantly less positive mood after direct gaze (*B*=-1.62, SE=0.67, t(77)=-2.41, *p*=0.018, *d*=0.55), but not after averted gaze (*p*=0.218). There was no significant interaction between CEM scores and target on participants’ mood responses (*p*=0.970), indicating that participants’ mood did not differ after videos of themselves versus an unfamiliar other. There was no significant three-way interaction between CEM scores, target, and gaze direction on participants’ mood responses (*p*=0.191). CEM was not significantly associated with participants’ self-reported mood, regardless of target and gaze direction (*p*=0.068).


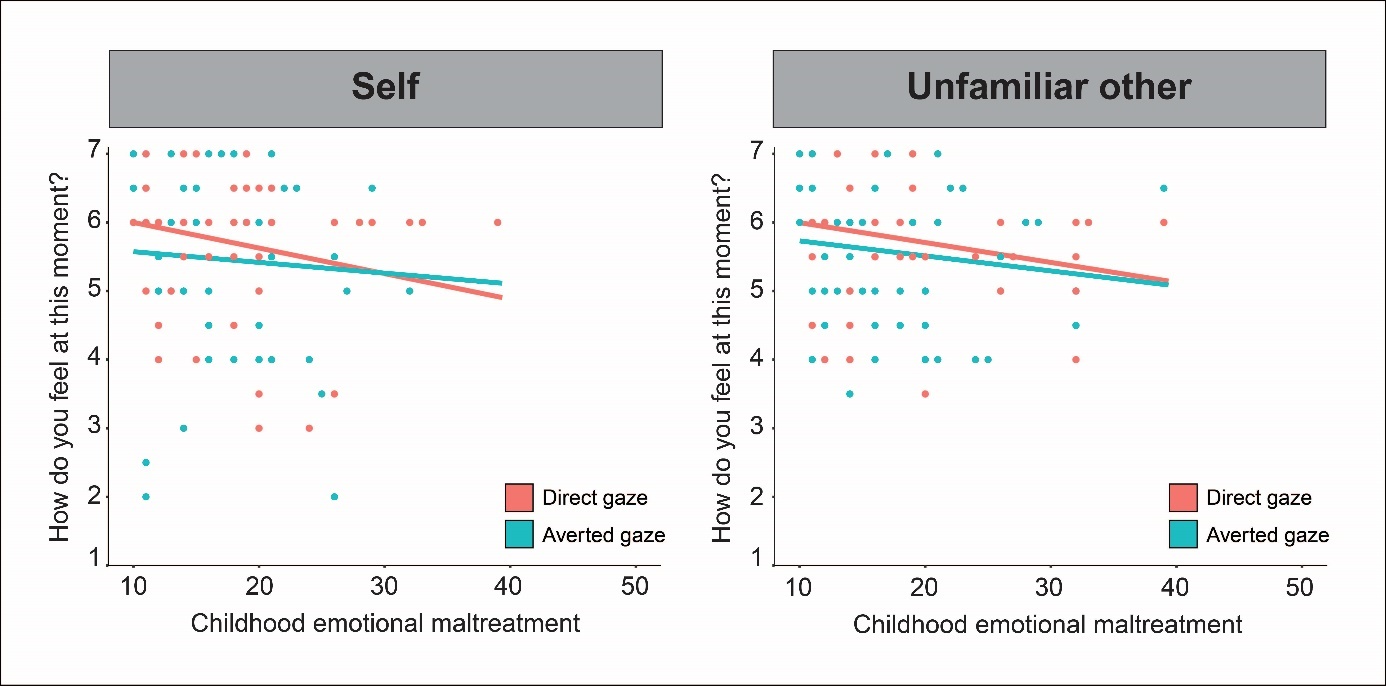


**Figure 2. Interaction between CEM and gaze direction (i.e., direct vs averted gaze) on participants’ self-reported mood after the videos of self and unfamiliar other.** Participants with more CEM experiences reported a significantly lower mood after direct, but not averted gaze, compared to participants fewer of such experiences (interaction CEM × gaze direction: *B*=0.67, SE=0.31, t(547)=2.20, *p*=0.028, *d*=0.19). There was no significant interaction between CEM and target (i.e., self vs. other) (*p* = .970), nor between CEM, gaze direction, and target on participants’ self-reported mood responses (*p* = .191).

*Gaze responses*

To examine whether CEM is associated with the percentage of dwell time towards the eyes of the self and an unfamiliar other, we performed a generalized linear mixed regression model with CEM, gaze direction (direct vs averted), and target (self vs unfamiliar other), and their interactions as predictors for the percentage of dwell time within the eye region of the targets (Figure 3). There were no significant interactions between CEM and target (*p*=0.168) or CEM and gaze direction (*p*=0.906), nor a significant three-way interaction between CEM, target, and gaze direction (*p*=0.220) on dwell time within the eye region of the targets. Also, CEM was not associated with participants’ dwell time within eye region, regardless of target or gaze direction (*p*=0.359).


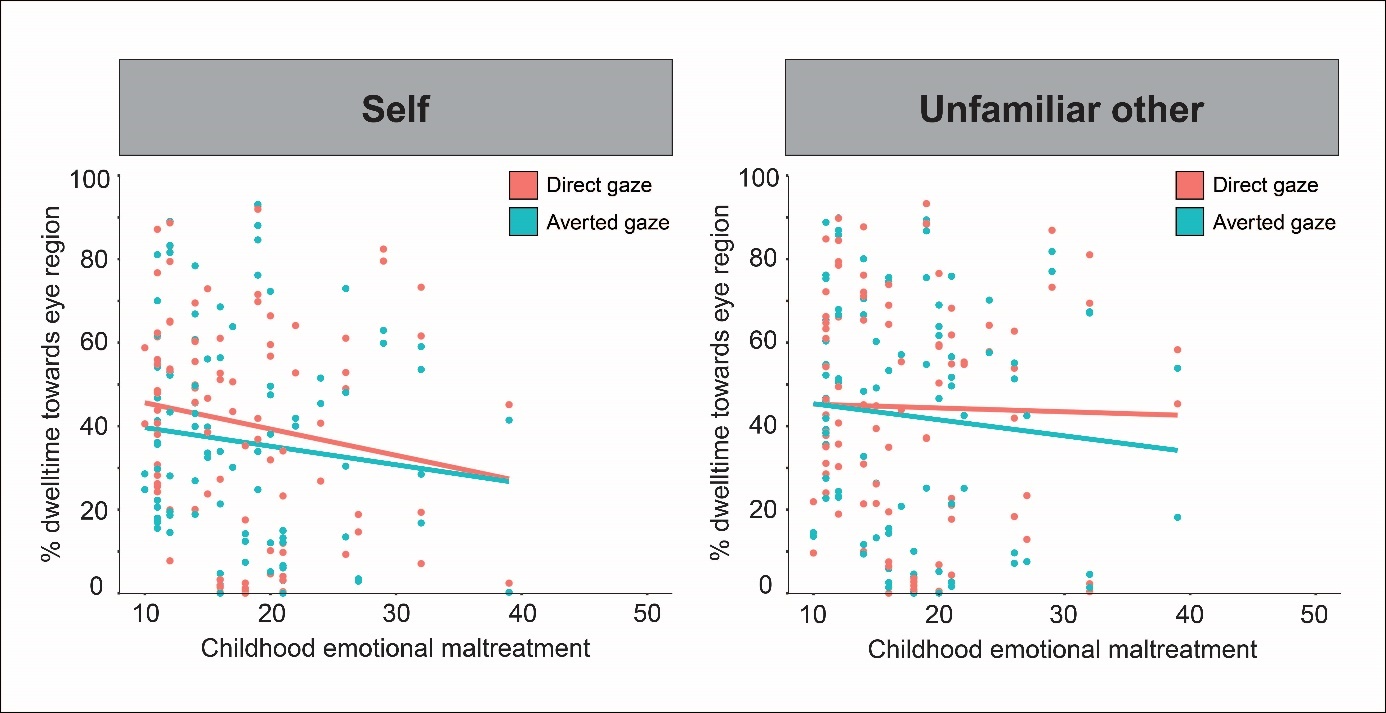


**Figure 3. Association between CEM and the percentage of dwell time within the eye region of targets relative to the total video duration for each gaze direction (i.e., direct vs averted gaze) during videos of self and an unfamiliar other.** There was no significant interaction between CEM and gaze direction (*p* = 0.906) or CEM and target (*p* = 0.168) on the percentage of dwell time within the eye region of targets.

*Neuroimaging results*

To test for associations between CEM and neural responses to direct minus averted gaze of the self and unfamiliar other, we performed two separate whole-brain regression analyses with participants’ experienced CEM scores as covariate of interest and their neural responses to direct gaze from either the self or unfamiliar other (Δdirect–averted gaze) as outcome variable. In response to participants’ own direct gaze, a significant positive association was found in the vmPFC (MNI-coordinate (14, 48, -8), *Z*=3.98, *k*=771, *p* _FWE-corr_=0.032 at cluster level), indicating that participants who reported more CEM showed enhanced BOLD-activation in vmPFC in response to their own direct versus averted gaze (Figure 4). In response to the gaze of an unfamiliar other person, we found no significant association between CEM and neural responses to direct (vs averted gaze). See Supplement 5 for main effect of target on participants’ neural responses during the task at whole-brain level. There was no main effect of gaze direction at the neural level.

**
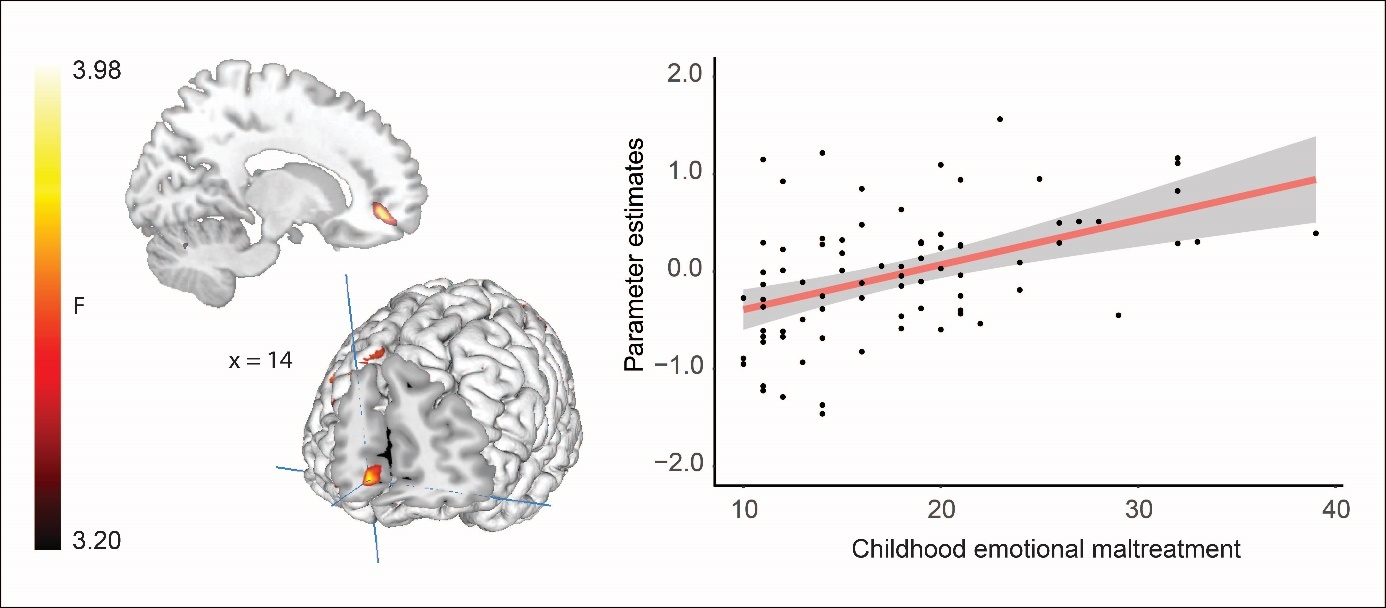
**

**Figure 4. CEM was associated with increased vmPFC activity when looking into one’s own eyes.** A whole-brain regression analysis testing for a positive association between CEM and neural responses to participants’ own direct gaze (Δ direct gaze – averted gaze) yielded a significant cluster in vmPFC (MNI-coordinate [14, 48, -8], *Z*=3.98, *k*=771, *p* _FWE-corr_=0.032 at cluster level). To visualize this association, we plotted parameter estimates in this region against self-reported CEM scores of participants. Regression lines are plotted for illustration purposes only. Whole-brain analyses were thresholded at *p*<0.05 (FSW cluster-corrected using a cluster-forming threshold of *p* <0.001).

*Covariate analyses*

Given the associations between CEM and self-esteem (*r*=-0.37, *p*<0.001) and symptom severity of anxiety (*r*=0.34, *p*=0.002) and depression (*r*=0.23, *p*=0.043) in the current sample (Supplement 6), we ran a set of analyses to elucidate whether these measures might mediate the reported associations. All analyses were also controlled for participants’ sex and neuroimaging analyses were additionally controlled for handedness (left/right) and psychotropic medication status (yes/no) (see Supplement 7 for detailed information about these measures).

Associations between CEM and participants’ mood responses remained significant after controlling for participants’ sex, anxiety- or depression symptom severity, and self-esteem. The association between CEM and enhanced vmPFC activation in response to participants’ own direct gaze remained significant after controlling for sex and handedness, but was no longer significant when separately controlling for psychotropic medication status, severity of anxiety or depression symptoms, or self-esteem levels. We used a method to examine the sequential contribution of each of the regressors and averaged them over all possible sequential orderings to calculate the relative importance of each regressor (Relaimpo package in R). This analysis showed that childhood emotional maltreatment explained most of the variance (82.12%) of the relationship between childhood emotional maltreatment levels and enhanced vmPFC activation. Self-esteem explained 10.49%, anxiety severity explained 4.72%, and depression severity explained 2.67%. Together this shows that variance in vmPFC to one’s own gaze is mostly driven by individual differences in childhood emotional maltreatment and to a lesser extent by self-esteem, depression, and anxiety.

To further explore whether our results could be explained by other types of childhood maltreatment (i.e., physical abuse, physical neglect, and sexual abuse) we ran non-preregistered analyses in which we included these types of childhood maltreatment as covariates in the generalized linear mixed regression models when testing for the associations between CEM and 1) mood and 2) gaze behavior. All reported relationships between CEM and mood and gaze behavior as reported above remained intact. Furthermore, we controlled for physical abuse, physical neglect, and sexual abuse in the whole-brain regression analyses between CEM and neural responses to gazing into one’s own and another person’s eyes. The association between vmPFC activation and childhood emotional maltreatment remained significant after adding physical abuse and sexual abuse to the model, suggesting that this effect could not be explained by other types of childhood maltreatment. However, adding physical neglect to the regression model did not result in clusters that survived correction for multiple comparisons. On the one hand, this might suggest that physical neglect is, at least partly, associated with the enhanced vmPFC activation when participants are gazing into their own eyes in the eye contact task. On the other hand, considering that the severity of childhood maltreatment in this sample was mostly composed of emotional abuse, emotional neglect, and physical neglect, and to a lesser extent of physical abuse and sexual abuse, it might suggest a general effect of childhood maltreatment. As this cannot be disentangled based on the data of the current study, it is of interest to focus on this matter in a larger sample including a more balanced prevalence of all types of childhood maltreatment.

*Exploratory analyses*

In addition, we explored whether participants’ self-reported mood, gaze, and neural responses were uniquely associated with emotional abuse and emotional neglect. See Supplement 8 for a detailed overview of the results of these exploratory analyses. It should be noted that these analyses were not pre-registered and thus exploratory in nature. Our sample size was not a priori determined to have sufficient power to detect these nuanced effects. The results of these analyses need to be interpreted in the light of these limitations.

**Discussion**

This study shows that people who report moderate to extreme levels of CEM show enhanced vmPFC responses to one’s own direct gaze, but not to the gaze of a stranger. CEM’s effects on mood generalized to eye contact with other people, showing that CEM was not only associated with lower mood after directly gazing into one’s own eyes, but also after gazing into another person’s eyes. CEM was not associated with participants’ gaze responses into one’s own eyes or the eyes of a stranger in neither of the gaze directions.

Increased vmPFC activity in response to participants’ own direct gaze fits into a larger literature robustly linking vmPFC activity to affective aspects of self-evaluation (Moran, Macrae, Heatherton, Wyland, & Kelley, 2006; Northoff et al., 2006; Talmon et al., 2021; van der Meer et al., 2010; Will et al., 2020; Will, Rutledge, Moutoussis, & Dolan, 2017). Given the vmPFCs general role in emotion-regulation and representing subjective value, increased vmPFC activity in participants reporting CEM might indicate an increased engagement of self-related emotion-regulation processes in response to their own direct gaze. As such, vmPFC hyperactivation might signal a potential neural phenotype related to (maladaptive) patterns of increased self-referential processing associated with CEM that is activated when being confronted with one’s own gaze. This is in line with a study of Talmon et al. (2021) who found enhanced default mode network activation, including the same vmPFC region, in people diagnosed with social anxiety disorder and who reported to have a history of CEM. Interestingly, they did not find this effect in people diagnosed with social anxiety disorder without a history of CEM. Hyperactivation in a similar area of the vmPFC [MNI-coordinate: 10, 44, -7] was also found in depressed patients (versus healthy controls) in response to judgements about whether or not personality traits described themselves (Yoshimura et al., 2010), in particular for negative traits. The overlap between the clusters found in patients with social anxiety disorder and depression, and the cluster co-varying with CEM in our sample suggests that this region may be involved in negative self-attributions, either explicitly or in a more implicit manner (i.e., when looking at the self). The associations between CEM and vmPFC activation did not remain significant when controlling for participants’ depression- and anxiety severity, psychotropic medication status, or self-esteem. As indicated by their individual correlations, these variables show an association with CEM, but are not directly related with vmPFC activation. Therefore, CEM seems to be uniquely associated with participants’ neural response to one’s own direct gaze.

While people reporting less or no CEM showed a higher mood in response to direct versus averted gaze, this was not the case for individuals reporting more CEM, suggesting that people who with CEM do not benefit as much from the mood-boosting effects of making eye contact as people who reported less or no CEM. We found no evidence of CEM specifically moderating mood responses to videos of the self versus others. This is in contrast with participants’ neural responses, which showed a unique association of CEM on self-related, but not other-related stimuli in our sample. Although the reason for this discrepancy is not entirely clear, it suggests that higher CEM levels do not result in differential mood responses when seeing oneself versus a stranger.

CEM did not affect people’s amount of gaze into one’s own or other’s eyes. Nor did CEM differentially affect how much people gazed into the eyes of the targets during direct versus averted gaze videos. This is not in line with our hypotheses and clinical observations that people reporting more CEM show a greater tendency to avoid eye contact (Krill, 2011; Wilkinson, 2010). A possible explanation for this discrepancy is that the stimuli used in the eye contact task were prerecorded videos instead of real-live mutual gaze encounters similar to the clinical observations. This is emphasized by studies showing that eye contact only elevated participants’ levels of arousal in case of real-life bidirectional eye contact (Hietanen, Peltola, & Hietanen, 2020; Jarick & Bencic, 2019). Future studies on eye contact during real-live interactions should elucidate whether people’s gaze towards one’s own or others eyes co-varies with people’s reported CEM levels while receiving real-time visual feedback from the eyes of oneself (e.g., via a mirror) and others.

Observed associations between CEM and participants’ neural responses is striking given that the average age of our current sample was ±50 years, suggesting that the impact of CEM is still discernable well into adulthood. Moreover, our sample was not recruited based on participants having extreme levels of CEM. Thus, reported associations between brain and mood with CEM cannot be explained by a selection bias and seem to be even present in people who experienced moderate levels of CEM. In addition to these strengths, our study also has limitations. CEM was retrospectively reported by the participants about their childhood, which makes it susceptible to response biases. However, studies suggest that underreporting CEM is more common than over-reporting (Maughan & Rutter, 1997), indicating that participants’ CEM levels in the current study are probably representative for, or on the lower end of, the level of CEM they were exposed to. Although results match well-established findings showing that people who experienced CEM might be more self-conscious and have enhanced negative self-attributions (Alloy et al., 2006; Gibb, 2002; Talmon et al., 2021; van Harmelen et al., 2010), we did not ask participants to report on their self-views (e.g., self-disgust, self-compassion) in response to their own direct and averted gaze. Therefore, we cannot pinpoint which psychological processes may have driven the increased neural reactivity in vmPFC. Future studies might benefit from assessing self-cognitions to examine how they mediate people’s responses to their own gaze. Due to technical challenges of measuring eye gaze in the scanner we successfully collected gaze data from 48 of the 79 participants and therefore the results regarding participants gaze responses should be interpreted in the light of this reduced sample size. Lastly, given that prior studies have shown unique effects of emotional abuse and emotional neglect on individuals social and emotional functioning (Milojevich, Norwalk, & Sheridan, 2019; Warmingham, Handley, Rogosch, Manly, & Cicchetti, 2019), it is of interest to examine these unique (neural) correlates of the impact of emotional abuse and neglect on participants’ responses to eye contact in a larger sample.

Our results show that childhood emotional maltreatment is associated with enhanced neural responses to one’s own gaze. This impact on self-related processing seemed more pronounced for automatic processes (i.e., neural responses) and may be associated with more spontaneous affective reactions in people reporting more emotional maltreatment when gazing into one’s own eyes (e.g., sadness or self-loathing). Future studies should investigate whether treatments that focus on the strengthening of participants’ self-views (e.g., mindfulness-based cognitive therapy focused on negative self-referential processing or self-esteem training (Korrelboom, Maarsingh, & Huijbrechts, 2012; Lovas & Schuman-Olivier, 2018)) are beneficial for people who experienced CEM and whether such therapeutic interventions are able to normalize their behavioral and neural responses when connecting with oneself.

**Declaration of competing interest**

None.

**Author contributions**

**Mirjam C.M. Wever**: Conceptualization, Methodology, Software, Investigation, Formal analysis, Writing – Original Draft, Visualization. **Lisanne A.E.M. van Houtum**: Conceptualization, Software, Investigation. **Loes H.C. Janssen**: Investigation. **Wilma G.M. Wentholt**: Investigation. **Iris M. Spruit**: Methodology, Software. **Marieke S. Tollenaar**: Conceptualization, Writing – Review & Editing, Supervision. **Geert-Jan Will**: Conceptualization, Methodology, Software, Investigation, Writing – Review & Editing, Supervision. **Bernet M. Elzinga:** Conceptualization, Writing - Review & Editing, Supervision, Funding acquisition.

**Acknowledgements**

We would like to thank all families that participated in the study for their time and dedication. Furthermore, we thank all students that contributed to the recruitment and data collection. This study was supported by a personal research grant from the Netherlands Organization for Scientific Research (http://www.nwo.nl/) awarded to B.E. (VICI; Unravelling the Impact of Emotional Maltreatment on the Developing Brain 453–15–006). G-J.W. was additionally funded by the European Union’s Horizon 2020 research and innovation program under the Marie Skłodowska-Curie grant agreement (No 707404) and the Sara van Dam z.l. Foundation, Royal Netherlands Academy of Arts & Sciences.

**Data/code availability statement**

The de-identified data, analysis scripts and materials for this study are available on DataverseNL and the MRI data are available on NeuroVault (<https://neurovault.org/collections/UMDSFUUL/>). All study measures, hypotheses, and analyses were preregistered at Open Science Framework prior to data analyses ([https://osf.io/54nky](https://osf.io/54nky/?view_only=b55315eee49348e0833ced3746ae763f)). For any questions or additional material, please contact the corresponding author.

**References**

Alloy, L. B., Abramson, L. Y., Smith, J. M., Gibb, B. E., & Neeren, A. M. (2006). Role of parenting and maltreatment histories in unipolar and bipolar mood disorders: Mediation by cognitive vulnerability to depression. *Clinical child and family psychology review, 9*(1), 23-64. doi:10.1007/s10567-006-0002-4

Arntz, A., & Wessel, I. (1996). Jeugd trauma vragenlijst [Dutch version of the childhood trauma questionnaire]. *The Netherlands*.

Ashburner, J. (2007). A fast diffeomorphic image registration algorithm. *Neuroimage, 38*(1), 95-113. doi:10.1016/j.neuroimage.2007.07.007

Baldwin, C. L. (1996). *Self-complexity and physiological responses to facial self-reflection: An investigation into women's self-image*: University of North Texas.

Bates, D., Maechler, M., Bolker, B., Walker, S., Christensen, R. H. B., Singmann, H., . . . Grothendieck, G. (2012). Package ‘lme4’. *CRAN. R Foundation for Statistical Computing, Vienna, Austria*.

Baugh, L. M., Cox, D. W., Young, R. A., & Kealy, D. (2019). Partner trust and childhood emotional maltreatment: The mediating and moderating roles of maladaptive schemas and psychological flexibility. *Journal of Contextual Behavioral Science, 12*, 66-73. doi:10.1016/j.jcbs.2019.02.001

Bernstein, D. P., Stein, J. A., Newcomb, M. D., Walker, E., Pogge, D., Ahluvalia, T., . . . Desmond, D. (2003). Development and validation of a brief screening version of the Childhood Trauma Questionnaire. *Child abuse & neglect, 27*(2), 169-190. doi:10.1016/S0145-2134(02)00541-0

Bodden, D. H., Bögels, S. M., & Muris, P. (2009). The diagnostic utility of the screen for child anxiety related emotional disorders-71 (SCARED-71). *Behaviour research and therapy, 47*(5), 418-425. doi:10.1016/j.brat.2009.01.015

Conty, L., George, N., & Hietanen, J. K. (2016). Watching Eyes effects: When others meet the self. *Consciousness and cognition, 45*, 184-197. doi:10.1016/j.concog.2016.08.016

D'Argembeau, A. (2013). On the role of the ventromedial prefrontal cortex in self-processing: the valuation hypothesis. *Frontiers in human neuroscience, 7*, 372. doi:10.3389/fnhum.2013.00372

Dannlowski, U., Stuhrmann, A., Beutelmann, V., Zwanzger, P., Lenzen, T., Grotegerd, D., . . . Bauer, J. (2012). Limbic scars: long-term consequences of childhood maltreatment revealed by functional and structural magnetic resonance imaging. *Biological psychiatry, 71*(4), 286-293. doi:10.1016/j.biopsych.2011.10.021

Denny, B. T., Kober, H., Wager, T. D., & Ochsner, K. N. (2012). A meta-analysis of functional neuroimaging studies of self-and other judgments reveals a spatial gradient for mentalizing in medial prefrontal cortex. *Journal of cognitive neuroscience, 24*(8), 1742-1752. doi:10.1162/jocn_a_00233

Egeland, B. (2009). Taking stock: childhood emotional maltreatment and developmental psychopathology. doi:10.1016/j.chiabu.2008.12.004

Emery, N. J. (2000). The eyes have it: the neuroethology, function and evolution of social gaze. *Neuroscience & Biobehavioral Reviews, 24*(6), 581-604. doi:10.1016/S0149-7634(00)00025-7

Erdem, C. (2019). *The Utilization of the Mirror Interview to Explore Attachment Patterns, Shame and Body Representations in Adult Men.* (Ph.D.), The New School, Ann Arbor. ProQuest Dissertations & Theses Global database. (22616739)

Franck, E., De Raedt, R., Barbez, C., & Rosseel, Y. (2008). Psychometric properties of the Dutch Rosenberg self-esteem scale. *Psychologica Belgica, 48*(1), 25-35. doi:10.5334/pb-48-1-25

Gibb, B. E. (2002). Childhood maltreatment and negative cognitive styles: A quantitative and qualitative review. *Clinical psychology review, 22*(2), 223-246. doi:10.1016/S0272-7358(01)00088-5

Gibb, B. E., Abramson, L. Y., & Alloy, L. B. (2004). Emotional maltreatment from parents, verbal peer victimization, and cognitive vulnerability to depression. *Cognitive Therapy and Research, 28*(1), 1-21. doi:10.1023/B:COTR.0000016927.18027.c2

Gilbert, R., Widom, C. S., Browne, K., Fergusson, D., Webb, E., & Janson, S. (2009). Burden and consequences of child maltreatment in high-income countries. *The lancet, 373*(9657), 68-81. doi:10.1016/S0140-6736(08)61706-7

Gobin, R. L., & Freyd, J. J. (2014). The impact of betrayal trauma on the tendency to trust. *Psychological Trauma: Theory, Research, Practice, and Policy, 6*(5), 505. doi:10.1037/a0032452

Hietanen, J. K. (2018). Affective eye contact: an integrative review. *Frontiers in Psychology, 9*, 1587. doi:10.3389/fpsyg.2018.01587

Hietanen, J. O., Peltola, M. J., & Hietanen, J. K. (2020). Psychophysiological responses to eye contact in a live interaction and in video call. *Psychophysiology, 57*(6), e13587. doi:10.1111/psyp.13587

Jarick, M., & Bencic, R. (2019). Eye contact is a two-way street: Arousal is elicited by the sending and receiving of eye gaze information. *Frontiers in Psychology, 10*, 1262. doi:10.3389/fpsyg.2019.01262

Kaufman, J., Birmaher, B., Brent, D., Rao, U., & Ryan, N. (1996). Kiddie-Sads-present and Lifetime version (K-SADS-PL). *Pittsburgh, University of Pittsburgh, School of Medicine.* . doi:10.1097/00004583-199707000-00021

Korrelboom, K., Maarsingh, M., & Huijbrechts, I. (2012). Competitive Memory Training (COMET) for treating low self‐esteem in patients with depressive disorders: A randomized clinical trial. *Depression and anxiety, 29*(2), 102-110. doi:10.1002/da.20921

Krill, W. E. (2011). *Gentling: A Practical Guide to Treating PTSD in Abused Children*: Loving Healing Press.

Kroenke, K., Spitzer, R. L., & Williams, J. B. (2001). The PHQ‐9: validity of a brief depression severity measure. *Journal of general internal medicine, 16*(9), 606-613. doi:10.1046/j.1525-1497.2001.016009606.x

Lemogne, C., Gorwood, P., Bergouignan, L., Pélissolo, A., Lehéricy, S., & Fossati, P. (2011). Negative affectivity, self-referential processing and the cortical midline structures. *Social Cognitive and Affective Neuroscience, 6*(4), 426-433. doi:10.1093/scan/nsq049

Lovas, D. A., & Schuman-Olivier, Z. (2018). Mindfulness-based cognitive therapy for bipolar disorder: A systematic review. *Journal of Affective Disorders, 240*, 247-261. doi:10.1016/j.jad.2018.06.017

Maughan, B., & Rutter, M. (1997). Retrospective reporting of childhood adversity: Issues in assessing long-term recall. *Journal of personality disorders, 11*(1), 19-33. doi:10.1521/pedi.1997.11.1.19

Milojevich, H. M., Norwalk, K. E., & Sheridan, M. A. (2019). Deprivation and threat, emotion dysregulation, and psychopathology: Concurrent and longitudinal associations. *Development and Psychopathology, 31*(3), 847-857.

Moran, J. M., Macrae, C. N., Heatherton, T. F., Wyland, C. L., & Kelley, W. M. (2006). Neuroanatomical evidence for distinct cognitive and affective components of self. *Journal of cognitive neuroscience, 18*(9), 1586-1594. doi:10.1162/jocn.2006.18.9.1586

Murray, R. J., Schaer, M., & Debbané, M. (2012). Degrees of separation: a quantitative neuroimaging meta-analysis investigating self-specificity and shared neural activation between self-and other-reflection. *Neuroscience & Biobehavioral Reviews, 36*(3), 1043-1059. doi:10.1016/j.neubiorev.2011.12.013

Northoff, G., Heinzel, A., De Greck, M., Bermpohl, F., Dobrowolny, H., & Panksepp, J. (2006). Self-referential processing in our brain—a meta-analysis of imaging studies on the self. *Neuroimage, 31*(1), 440-457. doi:10.1016/j.neuroimage.2005.12.002

Oldfield, R. C. (1971). The assessment and analysis of handedness: the Edinburgh inventory. *Neuropsychologia, 9*(1), 97-113. doi:10.1016/0028-3932(71)90067-4

Puetz, V., Viding, E., Maguire, E., Mechelli, A., Armbruster-Genç, D., Sharp, M., . . . McCrory, E. (2021). Functional brain plasticity following childhood maltreatment: A longitudinal fMRI investigation of autobiographical memory processing. *Development and Psychopathology*, 1-8.

R Core Team. (2013). R: A language and environment for statistical computing.

Revelle, W. (2012). Procedures for psychological, psychometric, and personality research. *Acesso em, 9*.

Reyome, N. D. (2010). Childhood emotional maltreatment and later intimate relationships: Themes from the empirical literature. *Journal of Aggression, Maltreatment & Trauma, 19*(2), 224-242. doi:10.1080/10926770903539664

Riggs, S. A. (2010). Childhood emotional abuse and the attachment system across the life cycle: What theory and research tell us. *Journal of Aggression, Maltreatment & Trauma, 19*(1), 5-51. doi:10.1080/10926770903475968

Rolls, E. T., Huang, C.-C., Lin, C.-P., Feng, J., & Joliot, M. (2019). Automated anatomical labelling atlas 3. *Neuroimage*, 116189. doi:10.1016/j.neuroimage.2019.116189

Rose, D., & Abramson, L. (1992). *Developmental predictors of depressive cognitive style: Research and theory In Cicchetti D & Toth S (Eds.), Rochester Symposium of Developmental Psychopathology*: University of Rochester Press, Rochester, NY.

Rosenberg, M. *Society and the adolescent self-image*: Vol. 11 (Princeton University Press, Princeton, 1965).

Sheehan, D. V., Lecrubier, Y., Sheehan, K. H., Amorim, P., Janavs, J., Weiller, E., . . . Dunbar, G. C. (1998). The Mini-International Neuropsychiatric Interview (MINI): the development and validation of a structured diagnostic psychiatric interview for DSM-IV and ICD-10. *The Journal of clinical psychiatry*. doi:10.1016/S0924-9338(97)83296-8

Somerville, L. H., Jones, R. M., Ruberry, E. J., Dyke, J. P., Glover, G., & Casey, B. (2013). The medial prefrontal cortex and the emergence of self-conscious emotion in adolescence. *Psychological Science, 24*(8), 1554-1562. doi:10.1177/0956797613475633

Spertus, I. L., Yehuda, R., Wong, C. M., Halligan, S., & Seremetis, S. V. (2003). Childhood emotional abuse and neglect as predictors of psychological and physical symptoms in women presenting to a primary care practice. *Child abuse & neglect, 27*(11), 1247-1258. doi:10.1016/j.chiabu.2003.05.001

Stoltenborgh, M., Bakermans‐Kranenburg, M. J., Alink, L. R., & van IJzendoorn, M. H. (2015). The prevalence of child maltreatment across the globe: Review of a series of meta‐analyses. *Child Abuse Review, 24*(1), 37-50. doi:10.1002/car.2353

Talmon, A., Dixon, M. L., Goldin, P. R., Heimberg, R. G., & Gross, J. J. (2021). Neurocognitive heterogeneity in social anxiety disorder: The role of self-referential processing and childhood maltreatment. *Clinical Psychological Science, 9*(6), 1045-1058.

Thombs, B. D., Bernstein, D. P., Lobbestael, J., & Arntz, A. (2009). A validation study of the Dutch Childhood Trauma Questionnaire-Short Form: factor structure, reliability, and known-groups validity. *Child abuse & neglect*. doi:10.1016/j.chiabu.2009.03.001

Tottenham, N., Hare, T. A., Millner, A., Gilhooly, T., Zevin, J. D., & Casey, B. (2011). Elevated amygdala response to faces following early deprivation. *Developmental science, 14*(2), 190-204. doi:10.1111/j.1467-7687.2010.00971.x

van der Meer, L., Costafreda, S., Aleman, A., & David, A. S. (2010). Self-reflection and the brain: a theoretical review and meta-analysis of neuroimaging studies with implications for schizophrenia. *Neuroscience & Biobehavioral Reviews, 34*(6), 935-946. doi:10.1016/j.neubiorev.2009.12.004

van Harmelen, A.-L., de Jong, P. J., Glashouwer, K. A., Spinhoven, P., Penninx, B. W., & Elzinga, B. M. (2010). Child abuse and negative explicit and automatic self-associations: the cognitive scars of emotional maltreatment. *Behaviour research and therapy, 48*(6), 486-494. doi:10.1016/j.brat.2010.02.003

van Harmelen, A.-L., van Tol, M.-J., Demenescu, L. R., van der Wee, N. J., Veltman, D. J., Aleman, A., . . . Elzinga, B. M. (2013). Enhanced amygdala reactivity to emotional faces in adults reporting childhood emotional maltreatment. *Social Cognitive and Affective Neuroscience, 8*(4), 362-369. doi:10.1093/scan/nss007

van Houtum, L. A., Wever, M. C., Janssen, L. H., van Schie, C. C., Will, G.-J., Tollenaar, M. S., & Elzinga, B. M. (2021). Vicarious praise and pain: parental neural responses to social feedback about their adolescent child. *Social Cognitive and Affective Neuroscience, 16*(4), 406-417. doi:10.1093/scan/nsab004

Van Steensel, F., & Bögels, S. (2014). An adult version of the screen for child anxiety related emotional disorders (SCARED-A). *Netherlands Journal of Psychology, 68*.

Vergallito, A., Mattavelli, G., Gerfo, E. L., Anzani, S., Rovagnati, V., Speciale, M., . . . Lauro, L. J. R. (2020). Explicit and implicit responses of seeing own vs. others’ emotions: An electromyographic study on the neurophysiological and cognitive basis of the self-mirroring technique. *Frontiers in Psychology, 11*, 433. doi:10.3389/fpsyg.2020.00433

Viola, P., & Jones, M. (2001). Rapid object detection using a boosted cascade of simple features. *Proceedings of the 2001 IEEE computer society conference on computer vision and pattern recognition. CVPR 2001, 1*, I-I. doi:10.1109/CVPR.2001.990517

Warmingham, J. M., Handley, E. D., Rogosch, F. A., Manly, J. T., & Cicchetti, D. (2019). Identifying maltreatment subgroups with patterns of maltreatment subtype and chronicity: A latent class analysis approach. *Child abuse & neglect, 87*, 28-39.

Wever, M. C., van Houtum, L. A., Janssen, L. H., Wentholt, W. G., Spruit, I. M., Tollenaar, M. S., . . . Elzinga, B. M. (2022). Neural and Affective Responses to Prolonged Eye Contact with One's Own Adolescent Child and Unfamiliar Others. *Neuroimage*, 119463. doi:10.1016/j.neuroimage.2022.119463

Wever, M. C., van Houtum, L. A., Janssen, L. H., Will, G.-J., Tollenaar, M. S., & Elzinga, B. M. (2021). Neural signatures of parental empathic responses to imagined suffering of their adolescent child. *Neuroimage, 232*, 117886. doi:10.1016/j.neuroimage.2021.117886

Wickham, H., Chang, W., & Wickham, M. H. (2016). Package ‘ggplot2’. *Create Elegant Data Visualisations Using the Grammar of Graphics. Version, 2*(1), 1-189.

Wilkinson, M. (2010). *Changing Minds in Therapy: Emotion, Attachment, Trauma, and Neurobiology (Norton Series on Interpersonal Neurobiology)*: WW Norton & Company.

Will, G.-J., Moutoussis, M., Womack, P. M., Bullmore, E. T., Goodyer, I. M., Fonagy, P., . . . Dolan, R. J. (2020). Neurocomputational mechanisms underpinning aberrant social learning in young adults with low self-esteem. *Translational psychiatry, 10*(1), 1-14. doi:10.1038/s41398-020-0702-4

Will, G.-J., Rutledge, R. B., Moutoussis, M., & Dolan, R. J. (2017). Neural and computational processes underlying dynamic changes in self-esteem. *Elife, 6*, e28098. doi:10.7554/eLife.28098

Wright, M. O. D., Crawford, E., & Del Castillo, D. (2009). Childhood emotional maltreatment and later psychological distress among college students: The mediating role of maladaptive schemas. *Child abuse & neglect, 33*(1), 59-68. doi:10.1016/j.chiabu.2008.12.007

Yoshimura, S., Okamoto, Y., Onoda, K., Matsunaga, M., Ueda, K., & Suzuki, S.-i. (2010). Rostral anterior cingulate cortex activity mediates the relationship between the depressive symptoms and the medial prefrontal cortex activity. *Journal of Affective Disorders, 122*(1-2), 76-85. doi:10.1016/j.jad.2009.06.017

Ypsilanti, A., Robson, A., Lazuras, L., Powell, P. A., & Overton, P. G. (2020). Self-disgust, loneliness and mental health outcomes in older adults: an eye-tracking study. *Journal of Affective Disorders, 266*, 646-654. doi:10.1016/j.jad.2020.01.166

**SUPPLEMENTARY MATERIAL**

**SUPPLEMENT 1**

***Procedure***

Participants were recruited via public advertisements, (online) social media, and mental health facilities. They were briefed about the study and underwent a comprehensive telephone screening during which in- and exclusion criteria were assessed, and informed consent was discussed. Inclusion criteria for families with an adolescent without psychopathology were: no diagnoses of any (neuro)psychiatric disorders in the two years leading up to the study and no lifetime diagnosis of MDD/dysthymia. Inclusion criteria for families with an adolescent with MDD/dysthymia were that adolescents met criteria for one of these current primary diagnoses, verified with the Kiddie-Schedule for Affective Disorders and Schizophrenia Present and Lifetime version (K-SADS; (Kaufman, Birmaher, Brent, Rao, & Ryan, 1996)). Families with an adolescent with MDD/dysthymia could not participate if the adolescent met criteria for a primary diagnosis of another current (neuro)psychiatric disorder or a comorbid psychosis, substance use disorder, or mental retardation. Additional exclusion criteria were incompatibilities with MRI scanning (e.g., metal implants, pregnancy). When eligible for participation, they were invited for two appointments: An assessment day in the lab and an MRI session on a separate day. Prior to the first appointment, participants were asked to fill out an online questionnaire battery including demographic and psychosocial measures, including childhood emotional maltreatment, anxiety- and depression severity, and state self-esteem. During the first appointment, they performed parent-adolescent interaction tasks together with their child and filled out additional questionnaires, and participants’ current Axis-I psychopathology based on DSM-IV was assessed with the full version of the semi-structured Mini International Neuropsychiatric Interview (M.I.N.I.; Dutch version 5.0.0), except for the optional module about antisocial personality disorders (Sheehan et al., 1998). The interview was taken by trained Psychology Master students who held the interview face-to-face. During the second appointment, participants underwent an MRI scan at the LUMC in Leiden, the Netherlands (median of days between the first and second appointment = 37; range 7-265 days). Prior to the scan, they filled out a set of questionnaires, received instructions about the MRI tasks, and performed some practice trials. Participants performed three tasks in the MRI scanner: the eye contact task, which is the focus of the current study (Wever et al. (2022) for analyses of task effects), a parental empathy task (Wever et al., 2021), and the vicarious social feedback task (van Houtum et al., 2021). Upon completion of the MRI scans, participants were fully debriefed about the goals of the study and received a monetary compensation for participation and travel expenses. Participants provided written informed consent for each individual testing day.

**SUPPLEMENT 2**

**Results regarding the association between CEM and participants’ mood, gaze, and neural responses to all targets (i.e., own child, unfamiliar child, unfamiliar adult, self).**

Data presented in the current manuscript were collected in the context of a larger study and a more extensive eye contact task including two additional condition, e.g., own child and unfamiliar child. General task effects on prolonged eye contact (i.e., direct versus averted gaze) towards others (i.e., own child, unfamiliar child and unfamiliar adult) and the self are described and published elsewhere (Wever et al., 2022). The current study focuses on associations between participants’ self-reported levels of experienced CEM and affective, gaze, and neural responses to eye contact of self and an unfamiliar other. For the sake of transparency and completeness, we additionally ran a 2 × 4 ANOVA model including all ‘other’ conditions of the original task (i.e., own child, unfamiliar child, unfamiliar adult) and the self on participants’ self-reported mood and gaze responses. In addition, we ran two separate whole-brain regression analyses to assess the association between participants’ experienced CEM scores and their neural responses to a prolonged direct gaze from their own child and an unfamiliar child. The results of these analyses are described below. Please note that we only had a priori hypotheses about the self and unfamiliar other contrast.

Overall, CEM was significantly associated with participants’ mood responses in general, showing that participants who experienced more CEM reported a significantly lower mood compared to participants with less experienced CEM (*B* = -1.36, SE = 0.63, t(77) = -2.16, *p* = 0.034, *d* = 0.49). There was no significant interaction between CEM and gaze direction (*p =* 0.065) or CEM and target (*p* = 0.883) on participants mood, indicating that this association was not moderated by the gaze direction or the identity of the targets in the videos. Also, the three-way interaction between CEM, target, and gaze direction on participants’ mood responses was not significant (*p* = 0.358).

CEM was not significantly associated with the percentage of gaze within the eye region of the targets (*p* = 0.428). Also, there was no significant interaction between CEM and gaze direction (*p* = 0.937) or CEM and target (*p* = 0.395), or the three-way interaction between CEM, target, and gaze direction (*p* = 0.522) on the extent to which participants gaze within the eye region of targets, indicating that this association was not moderated by gaze direction or target of the targets in the videos.

To test for associations between a CEM and neural responses to direct gaze (Δdirect minus averted gaze) from participants’ own child and an unfamiliar child (same sex as own child), we performed two separate whole-brain regression analyses with participants’ experienced CEM scores as predictor variable and their neural responses to direct gaze from either their own child or an unfamiliar child (Δdirect minus averted gaze) as outcome variable. These analyses yielded no significant associations between CEM and neural responses to direct gaze from either participants’ own child or an unfamiliar child.

**SUPPLEMENT 3**

**Stimuli development**

For the preparation of the video fragments, we recorded videos of participants during the separate assessment day in the lab. The videos had to have a minimal duration of 45 s and were recorded in front of a white wall. Participants were asked to wear a black t-shirt during the recordings in order to avoid distraction due to their clothing. We asked them to look directly into the camera with a friendly, but neutral, facial expression. They were also instructed not to stare, but to gaze as natural as possible and blinking was allowed. The targets depicting the unfamiliar other condition for both sexes were approached in the context of stimuli development for the current task and were selected based on age (between 45-55 years) and sex. Videos were recorded under similar circumstances as videos of the participants and written informed consent was taken to confirm the approval of the targets to use their videos in the current study.

**SUPPLEMENT 4**

**fMRI data acquisition**

MRI images were acquired at the LUMC using a Philips 3.0T Achieva MRI scanner equipped with a SENSE-32 channel head coil. For the eye contact task, T2*-weighted echo planar imaging (EPI) was used with the following parameters: TR = 2200 ms, TE = 30 ms, flip angle = 80°, FOV 220 × 220 × 114.7 mm, matrix size = 80 × 80, voxel size = 2.75 mm^3^, slice gap = 0.275 mm, 38 transverse slices in descending order. As the eye contact task was self-paced, number of volumes varied between participants (run 1: M = 152.6, SD = 12.2, range = 128 – 189; run 2: M = 149.1, SD = 12.3, range = 123 – 184). A structural 3D T1 scan was acquired with the following parameters: TR = 7.9 ms, TE = 3.5 ms, TI = 820 ms, flip angle: 8°, voxel size = 1 mm^3^, 155 transverse slices FOV 195.8 × 250 × 170.5 mm, matrix size = 228 × 177, duration: 4:11 min. The first five volumes were discarded to allow for equilibration of T1 saturation effects. A b0 field map was acquired with the following parameters: TR = 200 ms, TE = 3.2, matrix size = 80 × 80, with 38 slices, voxel size = 2.75 mm^3^.

Task responses were self-paced and participants could press any button to display a box around the middle option and then press the button corresponding to their right index (to go left) and right middle finger (to go right) to move the box to their preferred answer. They could confirm their answer by pressing the button corresponding to their left index finger.

The task was programmed and presented electronically and eye movement recordings were conducted using E-prime 2.0 (Tools Psychology Software, 2012) and participants could see the task through a mirror attached to the head coil. Foam inserts were used to restrict head motion if necessary. Scans were examined by a radiologist in case of any suspicion of abnormalities. Screen resolution was 1024 x 768 pixels. The videos were presented on the screen in 960 x 540 pixels. The task lasted approximately 11 minutes in total.

**SUPPLEMENT 5**

**Whole-brain peak activation coordinates for the self vs other and direct vs averted gaze contrasts.**

| Brain regions | MNI coordinates | | | Voxel test value | Cluster | Cluster |
| --- | --- | --- | --- | --- | --- | --- |
|  | **x** | **y** | **z** | **Z** | ***p*-value** | **size** |
| SELF > OTHER | | | | | | |
| R Middle occipital gyrus | 36 | -84 | 6 | >8 | <0.001 | 14022 |
| R Inferior temporal gyrus | 48 | -57 | -9 | 7.73 |  |  |
| R Superior parietal gyrus | 24 | -60 | 57 | 6.46 |  |  |
| L Middle occipital gyrus | -33 | -90 | 6 | 7.39 | <0.001 | 5807 |
| L Inferior occipital gyrus | -48 | -72 | -6 | 5.43 |  |  |
| L Inferior occipital gyrus | -56 | -77 | -9 | 4.94 |  |  |
| R Inferior frontal gyrus | 50 | 6 | 23 | 5.91 | 0.017 | 944 |
| R Insula | 33 | -3 | 17 | 3.90 |  |  |
| R Insula | 27 | -11 | 20 | 3.25 |  |  |
| R Inferior frontal gyrus | 53 | 42 | 8 | 5.70 | 0.006 | 1187 |
| OTHER > SELF | | | | | | |
| R Superior temporal gyrus (TPJ) | 60 | -56 | 21 | 7.08 | <0.001 | 6671 |
| R Angular gyrus | 53 | -47 | 30 | 6.17 |  |  |
| R Angular gyrus | 48 | -47 | 21 | 5.69 |  |  |
| L Superior temporal gyrus (TPJ) | -60 | -56 | 20 | 6.71 | <0.001 | 8708 |
| L Middle temporal gyrus | -62 | -41 | -3 | 6.26 |  |  |
| L Middle temporal gyrus | -69 | -51 | -3 | 5.13 |  |  |
| R Calcerine sulcus | 12 | -75 | 11 | 6.60 | <0.001 | 16445 |
| R Cuneus | 6 | -72 | 30 | 6.39 |  |  |
| L Cuneus | 0 | -75 | 35 | 6.32 |  |  |
| L Inferior frontal gyrus | -51 | 23 | 20 | 6.07 | <0.001 | 6629 |
| L Precentral gyrus | -39 | 8 | 45 | 5.50 |  |  |
| L Inferior orbitofrontal gyrus | -44 | 44 | -8 | 5.27 |  |  |
| R Middle temporal gyrus | 74 | -33 | -5 | 5.80 | <0.001 | 5029 |
| R Middle temporal gyrus | 56 | -27 | -9 | 5.45 |  |  |
| R Middle temporal gyrus | 54 | 2 | -23 | 5.08 |  |  |
| R Medial superior frontal gyrus (dmPFC) | 6 | 47 | 30 | 5.27 | <0.001 | 6785 |
| R Medial superior frontal gyrus | 2 | 35 | 41 | 4.49 |  |  |
| L Supplementary motor area | -5 | 0 | 57 | 4.33 |  |  |
| DIRECT GAZE > AVERTED GAZE |  |  |  |  |  |  |
| *No significant clusters* |  |  |  |  |  |  |
| AVERTED GAZE > DIRECT GAZE |  |  |  |  |  |  |
| *No significant clusters* |  |  |  |  |  |  |

*Note.* The Automated Anatomical Labeling atlas (AAL3) by Rolls, Huang, Lin, Feng, and Joliot (2019) was used to label the peak coordinates.

**SUPPLEMENT 6**

Correlations between participants’ self-reported childhood emotional maltreatment (abuse and neglect) and symptom severity of anxiety, depression, and self-esteem levels.

| Measures | 1. | 2. | 3. |
| --- | --- | --- | --- |
| 1. CEM composite score (CTQ-SF) | - | - | - |
| 1. Depression symptom severity (PHQ-9) | 0.23* | - | - |
| 1. Anxiety symptom severity (SCARED-A) | 0.34** | 0.58*** | - |
| 1. Self-esteem score (RSES) | -0.37*** | -0.63*** | -0.63*** |

*Notes. n* = 79. Significant *p*-values < 0.05 were indicated by *, *p* < 0.01 by **, and *p* < 0.001 by ***. All abovementioned measures were log-transformed to account for skewness.

**SUPPLEMENT 7**

**Assessment of covariates psychotropic medication status, handedness, severity of anxiety- and depression symptoms, and self-esteem levels.**

*Psychotropic medication.* Seven participants used psychotropic medication at the day of the scan session, i.e., Citalopram (SSRI, n = 5), Venlafaxine (SNRI, n = 1), Concerta (Methylphenidate, n = 1).

*Handedness.* Handedness was assessed after the scan by a modified 10-item version of the Edinburgh Handedness Inventory (EHI) developed by Oldfield (1971). The self-report questionnaire consists of ten questions about which hand is used during specific actions and answer categories were always left (−100), most times left (-50), both (0), most times right (50), and always right (100). Sum scores were calculated by the following formula for the laterality quotient (LQ): [R-L]/[R + L] ∗ 100 and could range from +100 (right-handedness in all tasks) to −100 (left-handedness in all tasks). To convert the continuous laterality quotient into a dichotomous variable of right- and left-handedness that was used to control for in analyses we used the cut-off score of zero with quotients > 0 indicating right-handedness and < 0 indicating left-handedness. In the current sample, 71 participants were right-handed (89.9%) and 8 participants were left-handed (10.1%).

*Anxiety severity.* Prior to the assessment day in the lab, the severity of participants’ anxiety symptoms was assessed with an adjusted version of the Screen for Child Anxiety Related Emotional Disorders for Adults (SCARED-A; Van Steensel and Bögels (2014)). This version was based on the original 71-item version of the SCARED (Bodden, Bögels, & Muris, 2009), and includes 31 items of the original 71 items that derived from the subscales panic disorder, generalized anxiety and social anxiety. Participants were asked to assess to what extent the sentences were reflecting their current situation. Answers were given on a Likert scale ranging from: Not true or hardly even true (0), somewhat true or sometimes true (1), and very true or often true (2). The sum score could range from 0-62, with higher scores representing a higher anxiety severity. The SCARED-A has been validated in a Dutch sample and internal consistency, convergent, and divergent validity are good (Van Steensel & Bögels, 2014). SCARED-A scores of ≥ 20 were indicated as within the clinical range. The internal consistency in the current sample was α = 0.91. Sum scores were log-transformed to account for skewness.

*Depression severity.* After the scan, participants’ severity of depressive symptoms was assessed with the Patient Health Questionnaire (PHQ-9) (Kroenke, Spitzer, & Williams, 2001). The questionnaire includes nine items corresponding with the nine symptoms of a major depressive disorder of the DSM-IV. Participants were asked to assess how much they were bothered by the symptoms over the past two weeks. Answers were given on a Likert scale ranging from: Not at all (0), several days (1), more than half of the days (2), and nearly every day (3). The sum score could range from 0-27, with higher scores representing a higher depression severity. The PHQ-9 has been validated and internal consistency and test-retest reliability are excellent and criterion, construct and external validity were established (Kroenke et al., 2001). PHQ-9 scores of ≥ 15 were indicated as within the clinical range. The internal consistency in the current sample was α = 0.89. Sum scores were log-transformed to account for skewness.

*Self-esteem levels.* Prior to the assessment day in the lab, participants’ global trait-like self-esteem levels were assessed with the Dutch version of the Rosenberg Self-esteem Scale (RSES) (Franck, De Raedt, Barbez, & Rosseel, 2008; Rosenberg). This 10-item questionnaire measures a person’s overall evaluation of his or her worthiness as a human being and answers were given on a Likert scale ranging from 0 (*strongly disagree*) to 3 (*strongly agree*). The sum score could range from 0-30, with higher scores representing a higher global self-esteem. Psychometric properties of the RSES were good (Franck et al., 2008). Internal consistency in the current sample was α = 0.91. Sum scores were log-transformed to account for skewness.

SUPPLEMENT 8

**Unique associations between emotional abuse and emotional neglect and participants’ self-reported mood, gaze, and neural responses.**

We exploratory tested whether participants’ self-reported mood, gaze, and neural responses were uniquely associated with emotional abuse and emotional neglect.

*Mood responses.* We performed two generalized linear mixed regression models. One with emotional abuse, and one with emotional neglect as independent variable, instead of the composite CEM score. The regression models further included gaze direction (i.e., direct vs averted) and target (i.e., self vs unfamiliar other), and their interactions as predictors for participants’ self-reported mood responses. We found a significant interaction between emotional neglect and gaze direction on participants’ mood responses. Individuals who reported more emotional neglect reported a lower mood in response to a direct, but not an averted gaze. We did not find an interaction between emotional neglect and target, indicating that participants’ mood was independent of the gazer (either self or unfamiliar other). There was also no significant three-way interaction between emotional neglect, gaze direction, and target, and emotional neglect was not significantly associated with participants’ mood.

While there was a main effect between emotional abuse and participants’ mood (B = -1.38, SE = 0.67, t(77) = -2.06, p = 0.042), indicating that more experienced emotional abuse was associated with a lower self-reported mood throughout the task, we did not find a significant interaction between emotional abuse and gaze direction, nor between emotional abuse and target. Also, there was no three-way interaction between emotional abuse, gaze direction, and target.

*Gaze responses.* We performed two additional generalized linear mixed regression models. One with emotional abuse, and one with emotional neglect as independent variable, instead of the composite CEM score. The regression models further included gaze direction (i.e., direct vs averted) and target (i.e., self vs unfamiliar other), and their interactions as predictors for participants’ gaze responses towards the eye region of the targets. We found a significant interaction between emotional abuse and target on the amount of gaze towards the eye region. Individuals who reported more emotional abuse gazed more towards the eyes of others, while one’s history of emotional abuse did not affect how much they gazed into their own eyes. We did not find an interaction between emotional abuse and gaze direction, indicating that participants’ gaze responses were independent of whether they were confronted with a direct or an averted gaze. There was also no significant three-way interaction between emotional neglect, gaze direction, and target, and no main effect of emotional abuse on participants’ gaze responses.

We did not find a significant interaction between emotional neglect and gaze direction, nor between emotional neglect and target. Also, there was no three-way interaction between emotional abuse, gaze direction, and target, and main effect between emotional neglect and participants’ gaze responses.

*Neural responses.* Lastly, we examined whether emotional abuse and emotional neglect were associated with participants’ neural responses to a direct (minus averted) gaze of oneself and of an unfamiliar other (∆direct – averted gaze contrast). We found that more emotional neglect was associated with enhanced neural activation in right precentral gyrus. There was no significant correlation between emotional abuse and participants’ neural responses in the eye contact task, to none of the conditions. In line with the findings from the regression analysis including CEM, there were no significant results for the association between emotional abuse or emotional neglect and participants’ neural responses to a direct (minus) averted gaze of an unfamiliar other.

The significant interaction between emotional neglect and gaze direction on participants’ mood is consistent with the results of analyses with the composite CEM score. These exploratory additional analyses seem to suggest that this specific effect may have been driven by emotional neglect and not, or to a lesser extent, by emotional abuse. In contrast, the significant interaction between emotional abuse and target on participants’ gaze responses is inconsistent with the results of analyses with the composite CEM score. The findings remained significant after controlling for all individual covariates, including all other childhood maltreatment types (i.e., gender, age, anxiety symptoms, depression symptoms, self-esteem, physical abuse, physical neglect, sexual abuse, emotional abuse, and emotional neglect). It should be noted that these analyses were not pre-registered and thus exploratory in nature. Our sample size was not a priori determined to have sufficient power to detect these nuanced effects. The results of these analyses need to be interpreted in the light of these limitations.
